# Association between smoking cessation and periodontitis progression: a systematic review and meta-analysis of prospective observational studies

**DOI:** 10.3389/froh.2026.1900393

**Published:** 2026-07-29

**Authors:** Yangyang Xie, Ya Ma, Zhen Geng, Hongfan Zhao, Xiaonan Li, Liwen Wang, Peijing Yan, Xiliang Jiang, Min Zhang

**Affiliations:** 1 School of Stomatology, Southern Medical University, Guangzhou, Guangdong, China; 2 The First Clinical Medical College of Gansu University of Chinese Medicine (Gansu Provincial Hospital), Lanzhou, Gansu, China; 3 Department of Science and Technology, Sichuan Provincial People's Hospital, University of Electronic Science and Technology of China, Chengdu, China; 4 First Clinical Medical College, Lanzhou University, Lanzhou, Gansu, China; 5 Clinical Research Center, Sichuan Provincial People's Hospital, University of Electronic Science and Technology of China, Chengdu, China; 6 Department of Stomatology, Sichuan Provincial People's Hospital, University of Electronic Science and Technology of China, Chengdu, China

**Keywords:** meta-analysis, periodontitis, periodontitis progression, prospective observational study, smoking cessation

## Abstract

**Aim:**

This systematic review and meta-analysis aimed to investigate the association between smoking cessation and periodontitis progression by comparing periodontal outcomes in patients who quit smoking vs. those who continued smoking.

**Methods:**

PubMed, Embase, and the Cochrane Library were searched for prospective studies (published up to March 2025) on smoking cessation and periodontitis progression, with ≥3 months' follow-up. The primary outcome was probing depth. R Studio 4.1.3 was used for random-effects meta-analyses, sensitivity, and subgroup analyses.

**Results:**

Six studies (*n* = 1,934) were included. Smoking cessation was associated with short-term improvements in PD at 3 months (SMD = −1.08, 95%CI = −1.84 to −0.31, *P* < 0.01) and 6 months (SMD = −1.50, 95%CI = −2.45 to −0.55, *P* < 0.01), as well as CAL at 3 months (SMD = −0.78, 95%CI = −1.39 to −0.16, *P* = 0.01). However, these associations were not sustained at 12 months (PD: *P* = 0.10; CAL: *P* = 0.27). No significant associations were observed for BOP or PI at any follow-up interval.

**Conclusion:**

Smoking cessation may be associated with modest short-term improvements in periodontal parameters, particularly PD and CAL. However, further high-quality studies with longer follow-up are warranted to confirm clinical relevance.

## 1 Introduction

Periodontitis Is a condition that adversely effects periodontal tissues, characterized by diverse clinical, microbial, and immunological characteristics (1–3). The susceptibility or predisposition to periodontitis is influenced by a multitude of factors that can guide host responses towards either protective or destructive outcomes, with environmental factors, such as smoking, being among them (4). Periodontitis has drawn global attention as a significant health burden, affecting approximately 11.2% of the world's population. Notably, among dentate adults, the prevalence of periodontitis was estimated to be approximately 62% between 2011 and 2020, positioning it as the sixth most prevalent human disease (5, 6). Consequently, controlling periodontitis recurrence and progression holds crucial clinical and public health implications.

The development and progression of periodontitis are influenced by a complex interplay between environmental factors and genetic predisposition. Among these, environmental factors—particularly smoking—have been consistently identified as more significant contributors to disease risk and severity (7). Over the past two decades, several studies have highlighted smokers as a prominent risk factor for periodontitis (8–11). Smoking is a well-established risk factor for periodontitis, and smoking cessation is considered an essential component of comprehensive periodontal treatment (12). Smoking typically subjects the body to a chronic inflammatory state over an extended period. Thus, smoking has the potential to induce the onset of periodontitis. Chronic exposure to tobacco smoke leads to alterations in host immune responses, increased oxidative stress, and dysregulation of the inflammatory cascade, all of which contribute to periodontal tissue destruction. Mechanistically, smoking induces vasoconstriction, impairs neutrophil function, and elevates proinflammatory cytokines such as IL-1β, IL-6, and TNF-α in gingival tissues and crevicular fluid (13, 14). These alterations collectively contribute to periodontal tissue breakdown and facilitate disease progression. The hypothesis that smoking cessation may attenuate periodontitis progression is biologically plausible, given the potential for partial reversal or normalization of these pathophysiological processes. Cessation of smoking has been associated with a gradual reduction in local and systemic inflammatory burden, recovery of immune cell functionality, and improved microvascular perfusion. Moreover, former smokers consistently demonstrate more favorable clinical outcomes in response to both surgical and non-surgical periodontal therapies when compared to current smokers (15–17).

While previous systematic reviews have explored the association between smoking cessation and periodontal outcomes, critical gaps remain. Earlier work has examined smoking cessation's effects on periodontal parameters in both natural teeth and dental implants (18), as well as differences in periodontitis progression between smokers and non-smokers (19). However, these reviews did not perform meta-analyses, weakening the robustness of their conclusions. One study (20) assessed smoking cessation's influence on periodontitis incidence and progression but did not isolate its specific impact on disease progression alone. To date, only one meta-analysis (21) has investigated this relationship, but its inclusion of just two prospective studies (*n* = 78) limited its statistical power. Since its publication, new high-quality prospective data (*n* = 400) have emerged (11, 22), justifying an updated evidence synthesis. Furthermore, while smoking cessation may exert distinct short-term vs. long-term effects on periodontitis progression, existing reviews have not addressed this key temporal distinction (23). No meta-analysis has specifically evaluated smoking cessation's influence on periodontitis progression alone, nor have prior syntheses accounted for potential differential effects over time.

To address these gaps, we conducted a systematic review and meta-analysis of prospective observational studies to evaluate the impact of smoking cessation on periodontitis progression compared to continued smoking. We further performed subgroup analyses to assess potential short-term and long-term effects, providing a more nuanced understanding of this critical clinical question.

## 2 Methods

### 2.1 Protocol and reporting guideline

The protocol of this study was registered at OSF (https://doi.org/10.17605/OSF.IO/3DHG9). This meta-analysis was performed according to the guidelines of the Preferred Reporting Items for Systematic Reviews and Meta-Analyses (PRISMA 2020 statement) (MOOSE) (24, 25).

### 2.2 Information sources and search strategy

We searched the PubMed, EMBASE, and Cochrane Library databases from database inception up to March 1, 2025，using the following MESH terms: ‘‘Periodontitis’’, ‘‘Chronic Periodontitis’’, ‘‘Periodontal Diseases’’, ‘‘Cigar Smoking’’, ‘‘Tobacco Smoking’’, ‘‘Cigarette Smoking’’, ‘‘Tobacco Products’’, ‘‘Tobacco’’, ‘‘Tobacco Use’’, and ‘‘Smoking’’. We checked the reference lists of all relevant studies and review articles for additional eligible studies. The full search strategy is provided in Supplementary Appendix 1.

### 2.3 Eligibility criteria

Studies that met the following criteria were included: (1) prospective observational study regarding the association between smoking cessation and the progression of periodontitis; (2) studies comparing patients who received smoking cessation regardless of the cessation modality (e.g., counseling or pharmacotherapy), with patients who continued smoking; (3) The primary outcome measurement was probing depth (PD). Secondary outcomes included clinical attachment level (CAL), bleeding on probing (BOP), and plaque index (PI). Oral measurements included alveolar bone loss (ABL), tooth loss (TL), plaque score (PS), and gingival inflammation (GI); (4) human study and a prospective observational study. Any measure of these outcomes was eligible for inclusion.

The exclusion criteria for studies were as follows: (1) patients with a history of periodontal treatment within the six months preceding the initiation of smoking cessation; (2) other forms of smoking, apart from traditional smoking, including cigars and some electronic tobacco; (3) articles in non-English or non-Chinese.

### 2.4 Selection process

Two investigators (YY X and HF Z) independently performed the study selection according to the predefined eligibility criteria. After duplicate records were removed, the screening process was conducted in two stages. In the first stage, titles and abstracts were screened to exclude clearly irrelevant studies. In the second stage, the full texts of potentially eligible studies were screened for eligibility. Any discrepancies were resolved through discussion and consensus.

### 2.5 Data collection process

The information obtained from the studies was as follows: (1) basic information about the study: first author, published year, and study design; (2) information pertaining to the smoking cessation: definition and measurement of smoking cessation, the specific smoking modality such as electronic cigarette; (3) characteristics related to the outcome: definition and criteria used to evaluate periodontitis progression was gathered, and the timing of outcome measurement was set (3 months, 6 months, 9 months, and 12 months); (4) effect size: mean and SD (standard deviation) of each outcome at baseline and end point. For all continuous outcome measurements, data on changes from baseline were extracted. Data extraction was independently conducted by the same two investigators. In cases of disagreement, discussions were held to achieve consensus. For studies reporting outcomes at multiple follow-up time points, data from each time point were extracted separately to facilitate subgroup analyses according to follow-up duration.

### 2.6 Risk of bias and evidence certainty assessment

Two investigators (YY X and HF Z) independently assessed the risk of bias of the included studies utilizing the Newcastle-Ottawa Scale for cohort studies (26) (Supplementary Appendix 2). This scale assigns a maximum of eight points for the quality of study selection (0–4 points), comparability (0–1points), and outcome (0–3points) (Supplementary Appendix 3). Studies scoring 7-8 points were considered to be of high quality. Any disagreements were resolved through discussion and consensus.

We used the Grading of Recommendations Assessment, Development and Evaluation (GRADE) approach to assess the certainty of all pooled outcomes as high, moderate, low or very low. Observational evidence automatically started at low with the ability to upgrade or downgrade. The criteria for upgrading the certainty of evidence included factors such as a large effect size, the presence of a dose–response relationship, and the possibility that confounding factors might attenuate the observed effect. In contrast, evidence may be decreased for several reasons, including study limitations, inconsistency of results, indirectness of evidence, imprecision, and reporting bias (27).

### 2.7 Statistical analysis

The statistical analyses were performed using R studio 4.1.3 with the meta package loaded. The primary outcome (PD) and all secondary periodontal outcomes (CAL, BOP, and PI) were treated as continuous variables. Accordingly, pooled effect estimates were calculated as standardized mean difference (SMD) with corresponding 95% confidence intervals (95% CIs). All included studies with sufficient data were eligible for inclusion in the respective syntheses. To assess heterogeneity, we used the heterogeneity variance parameter $\tau^{2}$, the $I^{2}$ statistics, and the chi-square test (28). Specifically, an $I^{2}$ value ranging from 0% to 50% was considered to indicate low heterogeneity, 50% to 75% indicated moderate heterogeneity, and 75%–90% was considered to indicate high heterogeneity (29). When conducting the meta-combination, we employed the random-effects model to evaluate the progression of periodontal disease in patients having quit smoking compared with those who continue to smoke.

Prediction intervals (PIs) were computed under a random-effects model (≥ 3 studies) to estimate the potential range of true effects in future studies, accounting for both between-study heterogeneity (*τ*²) and the uncertainty of the pooled effect (30). The 95% PI reflects the interval within which the effect size of a new study is expected to lie with 95% probability, assuming a similar population and design as the included studies. Restricted maximum likelihood (REML) was used to estimate *τ*².

Furthermore, we conducted subgroup analyses stratified by follow-up periods to identify potential sources of heterogeneity and comprehensively explore the effect of follow-up duration on the association between smoking cessation and periodontitis. To evaluate publication bias, a funnel plot and Egger’s test were conducted when the meta-analysis encompassed more than ten studies. Additionally, due to potential heterogeneity arising from inconsistent definitions of definition and measurement of smoking cessation, co-interventions, and timing of smoking cessation of included studies, we conducted sensitivity analyses excluding studies that: (1) reported non-standard definitions of cessation (e.g., ‘ex-smokers’, ‘former smokers’); or (2) reported measurements of smoking cessation (e.g., ‘‘questionnaire’’, ‘‘measuring CO content in expired air’’, ‘‘detecting cotinine concentrations in saliva’’); or (3) included co-interventions such as periodontal therapy post-cessation; or (4) reported smoking cessation prior to baseline. These sensitivity analyses aimed to assess the robustness of the overall results.

## 3 Results

### 3.1 Literature search

**Figure 1:**
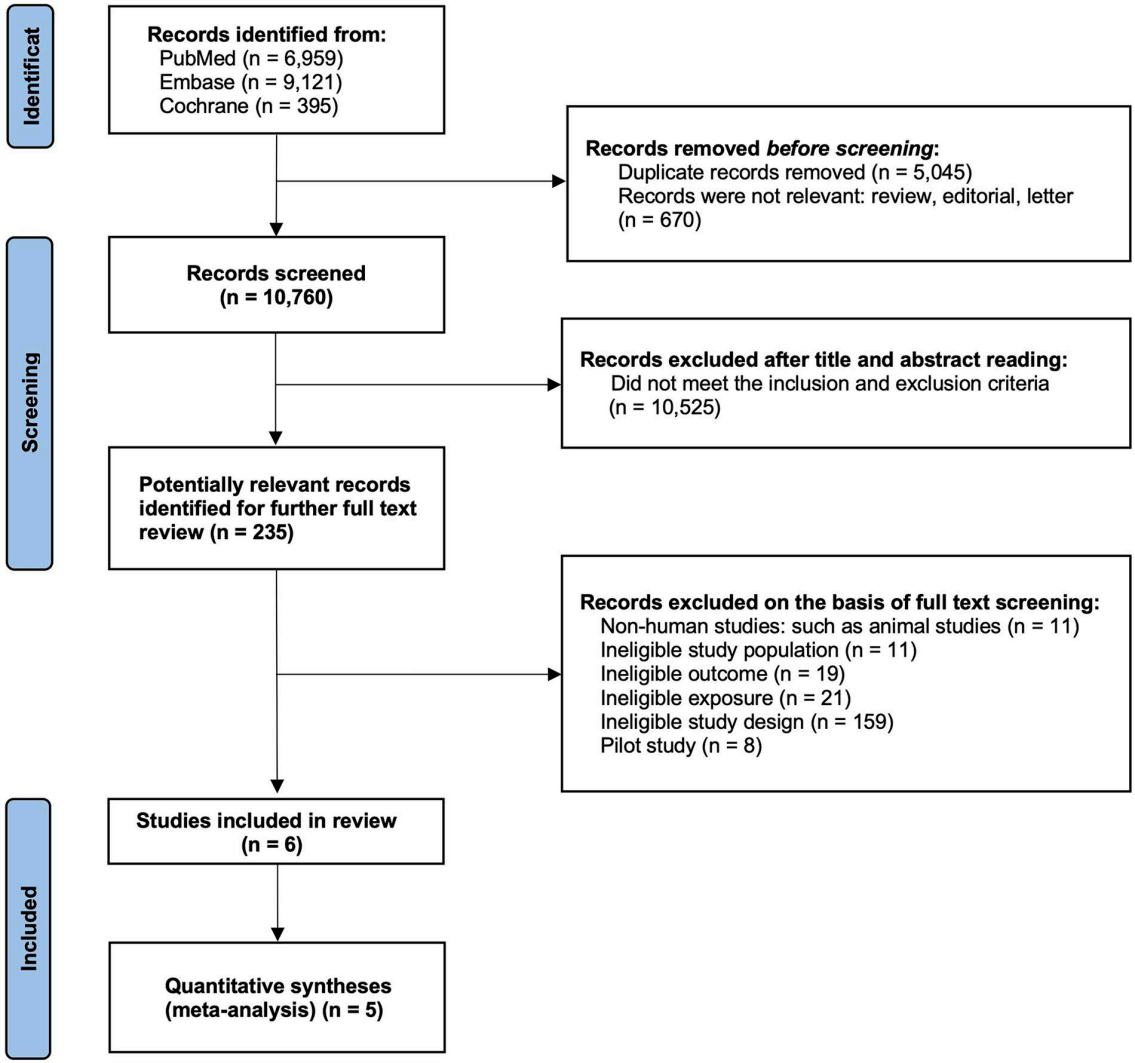
Selection of studies for systematic review and meta-analysis.

A flowchart illustrating the systematic review search process is presented in Figure 1. The initial search produced 16,475 results. After excluding 5,045 duplicate articles and 670 clearly irrelevant articles, 10,760 publications remained for review. Subsequently, upon screening the titles and abstracts, a further 10,525 articles were removed, leaving 235 articles for full-text reading (Supplementary Appendix 4). Upon conducting the full-text review, another 229 articles were excluded due to non-compliance with inclusion criteria. Consequently, the final review included a total of 6 prospective observational studies (11, 22, 31–34).

### 3.2 Characteristics of included studies

Six prospective observational studies, involving a total of 1,934 patients, were integrated into this research. The geographical scope of the study spanned across Asia, America, Europe and Africa, encompassing a significant portion of the global regions. The follow-up periods of the studies ranged from 6 months to 10 years. All the primary source studies had a follow-up longer than 12 months to ensure that patients had sufficient time to receive smoking cessation treatment. The primary approaches to smoking cessation included nicotine replacement therapy (NRT), cessation counselling and pharmacotherapy. The results were systematically presented according to different follow-up periods, including 3 months, 6 months, 9 months and 12 months. A comprehensive overview of the detailed characteristics of the included studies is provided in Table 1.

**Table 1:** Main characteristics of studies included in the systematic review and meta-analysis.

Year	First author	Country	Region	Follow-up duration (year)	Male (n, %)	Age (years) mean ± SD	Study design	Smoking cessation therapy	Co-interventions	Measurement of smoking cessation	Severity of periodontitis	Number of teeth (mean ± SD)	Comparison	Measurements	Main outcome	NOS score
2000	J. Bergström (31)	Sweden	Stockholm	10	89, 88.1	NR	Prospective study	NR	NR	Questionnaire	NR	NR	Former smokers vs. current smokers	per day	BOP, ABL, PI	7
2005	P. M. Preshaw (32)	UK	Newcastle	1	18, 36.7	42.0 ± 8.7	Prospective study	Cessation counselling; NRT; prescription of pharmaceuticals	Methodical root surface instrumentation; OHI	Diaries (self-reporting); CO content in expired air; cotinine concentrations in saliva	Moderate-to-severe chronic periodontitis	≥6 posterior teeth	Quitters vs. non-quitters	pack-year	PD, CAL, BOP, PS	7
2006	Y. Okamoto (33)	Japan	Nagoya	4	1332, 100.0	43.5 ± 6.4	Longitudinal Study	NR	NR	Questionnaire	NR	28.0 ± 2.5	Ex-smokers vs. smokers	per day	TL	8
2011	E. F. Rosa (34)	Brazil	Sa ˜o Paulo	1	20, 38.5	49.3 ± 6.7	Prospective study	Multi-disciplinary therapy; cessation lectures	Supra and subgingival scaling of all teeth; OHI; removal of all intraoral biofilm retentive factors	Questionnaire; monitoring of expired air CO	Destructive periodontal disease	≥10 teeth in the oral cavity	Quitters vs. non-quitters	pack-year	PD, CAL, BOP	7
2024	M. E. Rashid (11)	Saudi Arabia	Sakakah	0.5	NR	41.5 ± 11.8	Prospective study	Cessation counseling; pharmacotherapy	NR	NR	NR	NR	Quitters vs. non-quitters	Smoking history	PD, GI	8
2024	Arti Dixit (22)	India	Daman and Diu	1	NR	NR	Prospective study	Cessation counseling; NRT	NR	NR	Varying degrees of periodontal disease	NR	Quitters vs. non-quitters	Counseling sessions	PD, CAL, PI	8

NR, not reported; OHI, oral hygiene instructions; SD, standard deviation; NOS, Newcastle-Ottawa scale; PD, probing depth; BOP, bleeding on probing; ABL, alveolar bone loss; PI, plaque index; CAL, clinical attachment level; PS, plaque score; TL, tooth loss; GI, gingival index; NRT, nicotine replacement the.

### 3.3 Smoking cessation and progression of periodontitis

#### 3.3.1 Probing depth

**Figure 2:**
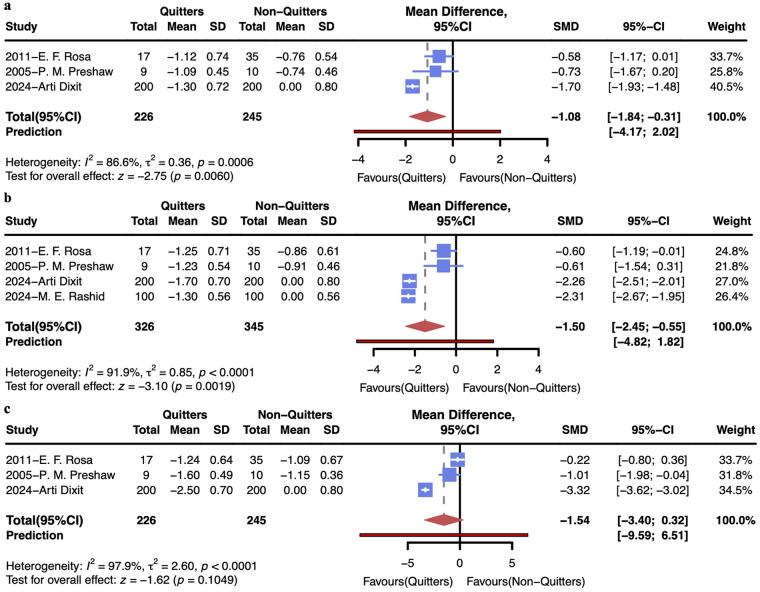
Forest plots for probing depth **(a)** 3-month group. **(b)** 6-month group. **(c)** 12-month group.

The meta-analysis on PD included four studies (*n* = 326) (Figure 2 and Supplementary Appendix 5**)**, which suggested that PD was associated with smoking cessation at follow-up periods of 3 months (SMD = −1.08, 95%CI = −1.84 – −0.31, *P* < 0.01, $I^{2}$ = 87%, $\tau^{2}$ = 0.36, P_her_ < 0.01, 95%PI = −10.22–88.07) and 6 months (SMD = −1.50, 95%CI = −2.45 – −0.55, *P* < 0.01, $I^{2}$ = 92%, $\tau^{2}$ = 0.85, P_her_ < 0.01, 95%PI = −5.99–2.99), based on very low certainty evidence. However, PD was not associated with smoking cessation at 12 months (SMD = −1.54, 95%CI = −3.40–0.32, *P* = 0.10, $I^{2}$ = 98%, $\tau^{2}$ = 2.60, P_her_ < 0.01, 95%PI = −25.31–22.23; very low certainty evidence).

#### 3.3.2 Clinical attachment level

**Figure 3:**
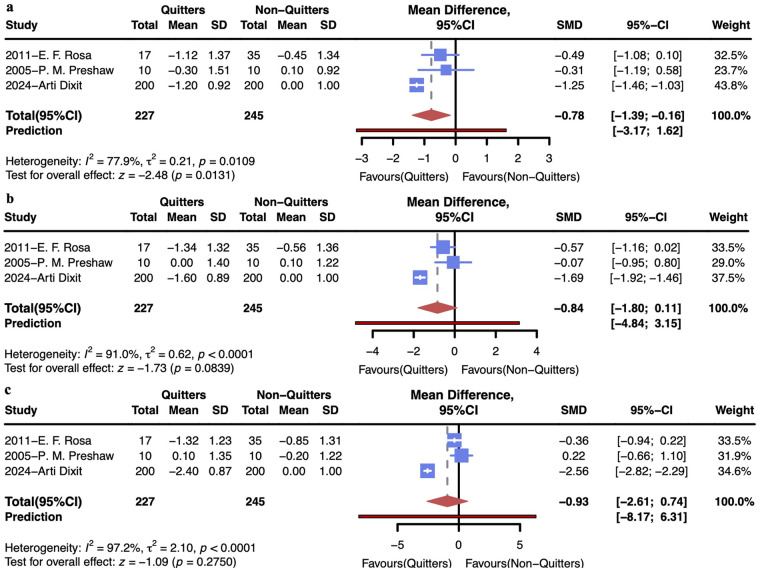
Forest plots for clinical attachment level **(a)** 3-month group. **(b)** 6-month group. **(c)** 12-month group.

The meta-analysis on CAL included three studies (*n* = 227) (Figure 3 and Supplementary Appendix 5**)**, showing that CAL was associated with smoking cessation at 3 months (SMD = −0.78, 95%CI = −1.39 to −0.16, *P* = 0.01, $I^{2}$ = 78%, $\tau^{2}$ = 0.21, P_he*r*_ = 0.01, 95%PI = −7.86–6.30; very low certainty evidence). However, CAL was not associated with smoking cessation at 6 months (SMD = −0.84, 95%CI = −1.80–0.11, *P* = 0.08, $I^{2}$ = 91%, $\tau^{2}$ = 0.62, P_her_ < 0.01, 95%PI = −12.64–10.95; very low certainty evidence) or 12 months (SMD = −0.93, 95%CI = −2.61–0.74, *P* = 0.27, $I^{2}$ = 97%, $\tau^{2}$ = 2.10, P_her_ < 0.01, 95%PI = −22.31–20.45; very low certainty evidence).

#### 3.3.3 Bleeding on probing

**Figure 4:**
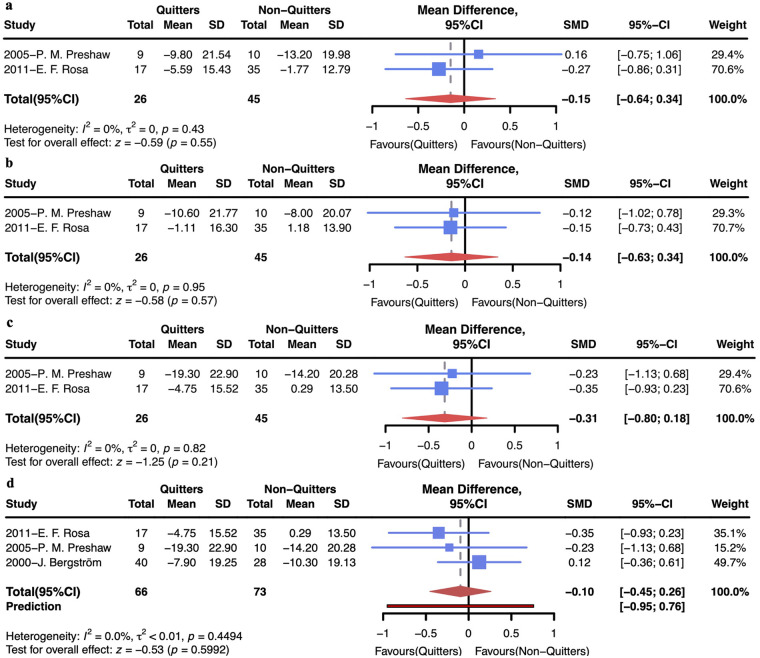
Forest plots for bleeding on probing **(a)** 3-month group. **(b)** 6-month group. **(c)** 12-month group. **(d)** Summary group.

The meta-analysis on BOP included three studies (*n* = 66) (Figure 4 and Supplementary Appendix 5), which indicated that BOP was not associated with smoking cessation (SMD = −0.10, 95%CI = −0.45–0.26, *P* = 0.60, $I^{2}$ = 0%, $\tau^{2}$ = 0.01, P_he*r*_ = 0.45, 95%PI = −2.63–2.44; very low certainty evidence). The subgroup analysis indicated that BOP was not associated with smoking cessation at follow-up periods of 3 months (SMD = −0.15, 95%CI = −0.64–0.34, *P* = 0.55, $I^{2}$ = 0%, $\tau^{2}$ = 0, P_he*r*_ = 0.43; very low certainty evidence), 6 months (SMD = −0.14, 95%CI = −0.63; 0.34, *P* = 0.57, $I^{2}$ = 0%, $\tau^{2}$ = 0, P_he*r*_ = 0.95; very low certainty evidence) and 12 months (SMD = −0.31, 95%CI = −0.80–0.18, *P* = 0.21, $I^{2}$ = 0%, $\tau^{2}$ = 0, P_he*r*_ = 0.82; very low certainty evidence).

#### 3.3.4 Plaque Index

**Figure 5:**
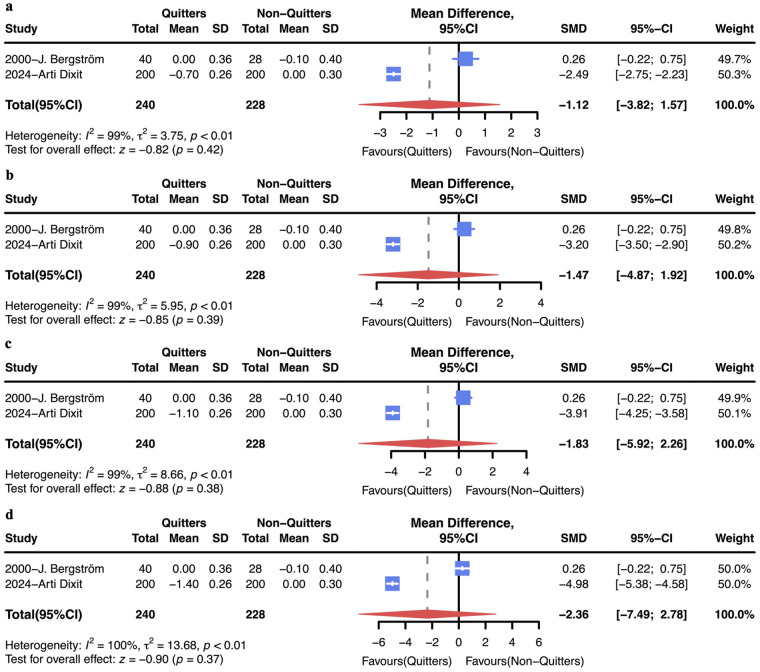
Forest plots for plaque index **(a)** 3-month group. **(b)** 6-month group. **(c)** 9-month group. **(d)** 12-month group.

The meta-analysis on clinical plaque index (PI) included two studies (*n* = 240) (Figure 5 and Supplementary Appendix 5), which indicated that PI was not associated with smoking cessation at follow-up periods of 3 months (SMD = −1.12, 95%CI = −3.82–1.57, *P* = 0.42, $I^{2}$ = 99%, $\tau^{2}$ = 3.75, P_her_ < 0.01; very low certainty evidence), 6 months (SMD = −1.47, 95%CI = −4.87–1.92, *P* = 0.39, $I^{2}$ = 99%, $\tau^{2}$ = 5.95, P_her_ < 0.01; very low certainty evidence), 9 months (SMD = −1.83, 95%CI = −5.92–2.26, *P* = 0.38, $I^{2}$ = 99%, $\tau^{2}$ = 8.66, P_her_ < 0.01; very low certainty evidence), and 12 months (SMD = −2.36, 95%CI = −7.49–2.78, *P* = 0.37, $I^{2}$ = 100%, $\tau^{2}$ = 13.68, P_her_ < 0.01; very low certainty evidence).

#### 3.3.5 Other secondary outcomes

The main feature of periodontitis is the alveolar bone loss (ABL). A long-term study (10 years) (31) indicated that within the long-term follow-up period, the mean alveolar bone height in the smoking group decreased conspicuously (mean change = −3.8), while in the smoking cessation group, it decreased slightly (mean change = −1.1). It was also clearly observed that the smoking cessation group had a smaller reduction in alveolar bone height and less alveolar bone resorption compared to the smoking group.

Tooth loss (TL) is one of the typical manifestations in the advanced stage of periodontitis. A long-term study (4 years) (33) reveals that the tooth loss in the smoking cessation group is substantially lower than that in the smoking group. Current smokers of ≥20 cigarettes per day had significantly higher TL risks compared with smoking cessation (20/day: OR = 2.01, 95%CI: 1.21-3.32; >21/day: OR = 2.06, 95%CI: 1.23-3.48), while no significant association was observed for light smokers (1-19/day: OR = 1.26, 95%CI: 0.60-2.64) or nonsmokers (OR = 1.11, 95%CI: 0.68-1.85).

Plaque score (PS) is an important indicator for measuring the quantity of plaque on the tooth surface. During the 12-month follow-up period (32), PS decreased significantly over time (*P* < 0.01), although no significant differences were observed between the smoking cessation and smoking groups (*P* = 0.36).

Gingival inflammation can reflect the severity of periodontitis. At the end of the 6-month follow-up (11), the GI score of the participants in the smoking cessation group decreased noticeably, suggesting a remarkable reduction in gingival inflammation. In contrast, the GI score of the smoking group did not show a discernible change throughout the entire study process.

#### 3.3.6 Sensitivity analyses

Sensitivity analyses were performed by omitting each study and calculating the pooled size to test the stability of the statistical results. This indicated that the results were relatively stable for PD (Supplementary Appendix 6), CAL (Supplementary Appendix 7), BOP **(**Supplementary Appendix 8), and PI (Supplementary Appendix 9). Regarding smoking cessation misclassification, among the six included studies, one defined cessation as ‘‘former smokers’’ (31) and another as ‘‘ex-smokers’’ (33), while the remaining used the term ‘‘quitters’’. A sensitivity analysis excluding the two studies with less standardized definitions showed stable results for PD, CAL, and PI (Supplementary Appendices 6–9**)**. Regarding the measurement of smoking cessation, a total of three measurement methods were involved: questionnaire, the measurement of CO content in expired air, and the determination of cotinine concentrations in saliva. In each meta-analysis, only two measurement approaches were included. After excluding one of them, the results demonstrated stability (Supplementary Appendices 6–9). Regarding co-intervention inconsistencies, two studies reported adjunctive treatments (e.g., periodontal debridement or oral hygiene instruction) after smoking cessation (32, 34). Excluding these two studies showed minimal impact, except for a slight attenuation in CAL at the 3-month follow-up (Supplementary Appendix 10). Two studies reported that smokers had quit smoking prior to baseline (31, 33): the former indicated a 9-year quit duration, while the latter did not specify the exact time, and the results remained stable despite this difference (Supplementary Appendices 6–9). These findings suggest that the results are robust to these sources of heterogeneity, although interpretative caution is warranted.

## 4 Discussion

To The best of our knowledge, this is a comprehensive systematic review and meta-analysis to evaluate the association between smoking cessation and the progression of periodontitis. The meta-analysis incorporated 6 prospective observational studies involving 1,934 participants. We found that smoking cessation has the potential to improve periodontal health.

Smoking has long been recognized as a significant risk factor for various health issues, including its pronounced effects on periodontal health. In particular, chronic smoking is strongly associated with the development of periodontitis (35–37). Several molecular mechanisms have been proposed for its pathogenesis: (1) smoking has been demonstrated to induce oxidative stress in periodontal tissues, leading to harmful effects (38); (2) nicotine, a component of tobacco smoke, has been associated with the upregulation of the expression of advanced glycation end-products (AGEs) accumulated in both periodontal soft and hard tissues (39); (3) smoking not only affects bacterial attack, periodontal tissue-host interactions, and immune-inflammatory responses, but also potentially worsens the progression of periodontitis and damages periodontal tissues by altering the microbial composition and host response factors (35, 32).

Taken together, these findings suggest that smokers are more susceptible to periodontitis and provide a biologically plausible rationale for considering smoking cessation as a potentially beneficial behavioral modification. It is estimated that smoking cessation programs can potentially reduce the incidence of destructive periodontal disease by approximately 12% in this population (34). Moreover, emerging evidence supports the adjunctive role of smoking cessation in the management of periodontitis (40–42). However, some of the proposed mechanisms—particularly those suggesting complete disease reversal or normalization of immune function following cessation—remain theoretical and lack direct empirical support from observational studies included in this review. These hypotheses should, therefore, be interpreted with caution and warrant further investigation through well-controlled experimental studies. A major limitation of the existing literature is the predominance of cross-sectional study designs, which restrict causal inference due to their inability to establish temporal relationships. In contrast, the present review synthesized data from prospective observational studies with consistent follow-up durations, thereby enabling a more robust pooled analysis.

While short-term improvements in periodontal clinical parameters—such as PD and CAL—were observed following smoking cessation, the evidence for sustained long-term benefits remains limited or inconclusive. This raises important questions regarding the durability of the therapeutic effect. One plausible explanation is that, in the absence of concomitant mechanical periodontal therapy (e.g., scaling and root planing), residual biofilm and calculus may persist and continue to drive inflammation and tissue destruction. This may attenuate or override the potential anti-inflammatory effects of smoking cessation over time (34). These observations suggest that the benefits of smoking cessation are likely adjunctive rather than curative when implemented in isolation. Accordingly, smoking cessation should be promoted as a critical component of an integrated periodontal treatment strategy (40), rather than as a replacement for active mechanical therapy. To better delineate these interactions, future research should prioritize long-term, well-controlled studies designed to disentangle the behavioral, biological, and clinical determinants of periodontal outcomes following smoking cessation.

Although no statistically significant differences were observed for BOP or PI, the pooled effect estimates consistently favored smoking cessation. For BOP, negative SMDs in both the overall and subgroup analyses suggested a trend toward reduced gingival inflammation after smoking cessation, although the available evidence was insufficient to confirm a significant effect. Likewise, although PI did not differ significantly between groups, the pooled estimates suggested lower plaque accumulation among individuals who quit smoking. Together, these findings may indicate gradual improvements in periodontal inflammatory status and oral hygiene behaviors following smoking cessation; however, they should be interpreted cautiously because of the limited number of studies and substantial heterogeneity. Several factors may explain this temporal pattern. First, consistent with previous studies (34), the short-term improvement in CAL may primarily reflect the resolution of gingival inflammation and tissue swelling rather than true periodontal attachment gain (23). In addition, because smoking suppresses the clinical signs of gingival inflammation (43). the restoration of normal inflammatory and vascular responses after smoking cessation may increase the clinical expression of inflammation despite an overall improvement in periodontal tissue health (13). Furthermore, without adequate mechanical periodontal therapy, persistent subgingival biofilm and calculus may continue to drive disease progression, thereby diminishing the long-term benefits of smoking cessation (23). Finally, maintaining smoking abstinence is challenging, and depressive symptoms associated with smoking cessation may adversely affect oral hygiene behaviors, potentially attenuating its long-term periodontal benefits (34).

Heterogeneity was a notable feature across most analyses in this review, with I² values often exceeding 85%. We explored possible sources of heterogeneity, including differences in follow-up duration, definitions of “quitters,” co-intervention, periodontal disease classification, and smoking cessation modalities. Specifically: 1) cessation methods ranged from NRT to counseling and pharmaceutical interventions, but varied too widely to permit consistent subgrouping; 2) Periodontal diagnoses spanned from moderate to destructive periodontitis, and in some cases, diagnostic criteria were not clearly stated; 3) Definitions of “quitters” varied—some studies used “former smokers,” others “ex-smokers” or simply “quitters”—potentially affecting comparability; 4) Studies comparing smokers who quit during periodontal therapy with those who did not, as well as studies investigating baseline smoking status unrelated to treatment, exhibit substantial differences in study quality. Such discrepancies may lead to variations in the timing of smoking cessation and its clinical implications.

Although we applied a random-effects model and calculated prediction intervals to account for between-study variability, these approaches cannot fully overcome the substantial clinical and methodological heterogeneity among the included studies. In addition, the small number of studies included in several subgroup analyses, particularly those stratified by follow-up duration (3, 6, and 12 months), further limits the precision and generalizability of our findings. Therefore, the pooled estimates should be interpreted with caution.

The strength of this meta-analysis lies in its comprehensive integration of diverse outcome measures, encompassing periodontal parameters such as PD and CAL, and clinical indices such as BOP and PI. However, our study has certain limitations. Firstly, due to the lack of relevant randomized controlled trials (RCTs), we exclusively included observational studies, which are naturally prone to confounding factors. Although we restricted inclusion to prospective studies to improve the reliability of the evidence, potential confounding factors, including periodontal disease classification, smoking exposure (e.g., cigarettes per day, pack-years, or nicotine dependence), smoking cessation modalities, duration of smoking cessation, and variations in the definition of smoking cessation, could not be further explored because these data were inconsistently reported across the included studies. Secondly, substantial heterogeneity was observed across several analyses. Although subgroup analyses based on follow-up duration, follow-up duration was not identified as a major source of heterogeneity. Thirdly, three distinct definitions of smoking cessation were identified among the included studies, namely “former smokers,” “ex-smokers,” and “quitters.” The first two definitions may have limitations due to the relatively early publication of the corresponding studies, during which the definition of smoking cessation had not yet been standardized. Finally, the sample size was not sufficiently large and exhibited substantial variability, especially for assessing long-term effects. Therefore, further studies with larger samples and longer follow-up periods are required to provide additional validation of the long-term association between smoking cessation and periodontitis.

## 5 Conclusions

Smoking cessation appears to be associated with modest short-term improvements in periodontal parameters, such as probing depth (PD) and clinical attachment level (CAL). However, current evidence is insufficient to establish a causal relationship or therapeutic effect, particularly in the absence of standardized periodontal interventions. Given the heterogeneity of smoking cessation definitions, observational study designs, and very low certainty of evidence (GRADE), these associations should be interpreted with caution. Notably, owing to the heterogeneity of intervention measures and the inadequate control of confounding factors, the clinical applicability of these findings remains limited. Further investigation is needed to validate the actual impact of smoking cessation. While smoking cessation may represent a potentially modifiable behavioral factor relevant to periodontitis management, its role should be understood as potentially adjunctive rather than independently efficacious. High-quality randomized controlled trials are needed to further evaluate these associations and clarify the potential mechanisms involved.

## Data Availability

The original contributions presented in the study are included in the article/Supplementary Material, further inquiries can be directed to the corresponding authors.
