# Supplementary material for: Association between smoking cessation and periodontitis progression: a systematic review and meta-analysis of prospective observational studies
Source: Front Oral Health. 2026 Jul 29;7:1900393. doi: 10.3389/froh.2026.1900393 (PMC13461921; doi:10.3389/froh.2026.1900393)
Supplement: Supplementary file 1 [file Datasheet1.docx]

***Appendix 1.*** Databases and search strategy for meta-analysis

| Database | Search terms or strategy |
| --- | --- |
| PubMed | (((((((Periodontitides) OR (Pericementitis)) OR (Pericementitides)) OR ("Periodontitis"[Mesh])) OR ((((((((Chronic Periodontitides) OR (Periodontitides, Chronic)) OR (Periodontitis, Adult)) OR (Periodontitis, Chronic)) OR (Adult Periodontitis)) OR (Adult Periodontitides)) OR (Periodontitides, Adult)) OR ("Chronic Periodontitis"[Mesh]))) OR (((((((Disease, Periodontal) OR (Diseases, Periodontal)) OR (Periodontal Disease)) OR (Parodontosis)) OR (Parodontoses)) OR (Pyorrhea Alveolaris)) OR ("Periodontal Diseases"[Mesh]))) AND (((((((((((((((((((((((((((((((((((((((((((((((((((((((((Smoking Behaviors) OR (Behavior, Smoking)) OR (Behaviors, Smoking)) OR (Smoking Behavior)) OR (Smoking Habit)) OR (Habit, Smoking)) OR (Habits, Smoking)) OR (Smoking Habits)) OR (Tobacco Uses)) OR (Tobacco Consumption)) OR (Consumption, Tobacco)) OR (Tobacco Chewing)) OR (Tobaccos)) OR (Nicotiana)) OR (Nicotianas)) OR (Nicotiana tabacum)) OR (Nicotiana tabacums)) OR (tabacum, Nicotiana)) OR (Product, Tobacco)) OR (Products, Tobacco)) OR (Tobacco Product)) OR (Cigarillos)) OR (Cigarillo)) OR (Pipe Tobacco)) OR (Pipe Tobaccos)) OR (Tobacco, Pipe)) OR (Tobaccos, Pipe)) OR (Cigars)) OR (Cigar)) OR (Kreteks)) OR (Kretek)) OR (Kreteks Tobacco)) OR (Kreteks Tobaccos)) OR (Tobacco, Kreteks)) OR (Tobaccos, Kreteks)) OR (Bidis)) OR (Bidi)) OR (Bidis Tobacco)) OR (Bidis Tobaccos)) OR (Tobacco, Bidis)) OR (Tobaccos, Bidis)) OR (Tobacco, Bidi)) OR (Bidi Tobacco)) OR (Bidi Tobaccos)) OR (Tobaccos, Bidi)) OR (Cigarettes)) OR (Cigarette)) OR (Smoking, Cigarette)) OR (Smoking, Tobacco)) OR (Smoking, Cigar)) OR ("Cigar Smoking"[Mesh])) OR ("Tobacco Smoking"[Mesh])) OR ("Cigarette Smoking"[Mesh])) OR ("Tobacco Products"[Mesh])) OR ("Tobacco"[Mesh])) OR ("Tobacco Use"[Mesh])) OR ("Smoking"[Mesh]))) |
| Cochrane | #1 MeSH descriptor: [Periodontitis] explode all trees 3483  #2 MeSH descriptor: [Chronic Periodontitis] explode all trees 972  #3 MeSH descriptor: [Periodontal Diseases] explode all trees 7845  #4 Periodontitides or Pericementitides or Pericementitis or Periodontitides, Adult or Periodontitides, Chronic or Adult Periodontitis or Chronic Periodontitides or Periodontitis, Adult or Periodontitis, Chronic or Adult Periodontitides or Disease, Periodontal or Diseases, Periodontal or Parodontoses or Periodontal Disease or Parodontosis or Pyorrhea Alveolaris 7834  #5 #1 or #2 or #3 or #4 11985  #6 MeSH descriptor: [Smoking] explode all trees 7569  #7 MeSH descriptor: [Tobacco Use] explode all trees 502  #8 MeSH descriptor: [Tobacco] explode all trees 280  #9 MeSH descriptor: [Tobacco Products] explode all trees 639  #10 MeSH descriptor: [Cigarette Smoking] explode all trees 232  #11 MeSH descriptor: [Tobacco Smoking] explode all trees 388  #12 MeSH descriptor: [Cigar Smoking] explode all trees 3  #13 Habits, Smoking or Habit, Smoking or Smoking Habit or Smoking Habits or Smoking Behaviors or Behavior, Smoking or Smoking Behavior or Behaviors, Smoking or Tobacco Chewing or Consumption, Tobacco or Tobacco Uses or Tobacco Consumption or Nicotiana or Tobaccos or Nicotianas or Nicotiana tabacum or Nicotiana tabacums or tabacum, Nicotiana or Tobacco, Pipe or Tobaccos, Pipe or Pipe Tobaccos or Pipe Tobacco or Cigarettes or Cigarette or Cigar or Cigars or Bidis Tobaccos or Bidis or Tobaccos, Bidi or Bidi or Bidi Tobacco or Tobacco, Bidis or Bidis Tobacco or Bidi Tobaccos or Tobaccos, Bidis or Tobacco, Bidi or Product, Tobacco or Products, Tobacco or Tobacco Product or Kreteks or Tobaccos, Kreteks or Kreteks Tobacco or Kretek or Kreteks Tobaccos or Tobacco, Kreteks or Cigarillos or Cigarillo or  Smoking, Cigarette or Smoking, Tobacco or Smoking, Cigar 23442  #14 #6 or #7 or #8 or #9 or #10 or #11 or #12 or #13 25556  #15 #5 and #14 407 |
| Embase | ('periodontitis'/exp OR 'paradontitis' OR 'parodontitis' OR 'peridontitis' OR 'periodontitis' OR 'chronic periodontitis'/exp OR 'adult periodontitis' OR 'chronic periodontitis' OR 'periodontitis chronica' OR 'periodontal disease'/exp OR 'dental loss' OR 'dental migration' OR 'dental mobility' OR 'furcation defects' OR 'mesial movement of teeth' OR 'paradontal disease' OR 'paradontopathy' OR 'paraodontopathy' OR 'parodentopathy' OR 'parodontal disease' OR 'parodontium disease' OR 'parodontive tissue disease' OR 'peridontal disease' OR 'peridontal tissue disease' OR 'peridontium disease' OR 'periodontal atrophy' OR 'periodontal attachment loss' OR 'periodontal disease' OR 'periodontal diseases' OR 'periodontal infection' OR 'periodontium disease' OR 'periodontopathy' OR 'tooth loss' OR 'tooth migration' OR 'tooth mobility' OR 'tooth movement') AND ('smoking'/exp OR 'behavior, smoking' OR 'behaviour, smoking' OR 'reverse smoking' OR 'smoker' OR 'smokers' OR 'smoking' OR 'smoking behavior' OR 'smoking behaviour' OR 'tobacco smoking' OR 'tobacco use'/exp OR 'tobacco usage' OR 'tobacco use'OR 'tobacco consumption'/exp OR 'tobacco chewing' OR 'tobacco consumption' OR 'tobacco'/exp OR 'condensate, tobacco' OR 'tobacco' OR 'tobacco condensate' OR 'tobacco constituent' OR 'tobacco product' OR 'tobacco products' OR 'tobacco smoke condensate' OR 'tobacco smoke extract' OR 'tobacco smoke residue' OR 'cigarette smoking'/exp OR 'cigarette smoker' OR 'cigarette smoking' OR 'smoking, cigarette' OR 'cigar smoking'/exp OR 'cigar smoke' OR 'cigar smoking' OR 'smoking, cigar') |
