# Supplementary material for: Association between smoking cessation and periodontitis progression: a systematic review and meta-analysis of prospective observational studies
Source: Front Oral Health. 2026 Jul 29;7:1900393. doi: 10.3389/froh.2026.1900393 (PMC13461921; doi:10.3389/froh.2026.1900393)
Supplement: Supplementary file 2 [file Datasheet10.docx]

***Appendix 10.*** **Sensitivity analysis comparison**

| Outcome measurements | Before omitting | | After omitting | |
| --- | --- | --- | --- | --- |
|  | SMD | 95% CI | SMD | 95% CI |
| PD |  |  |  |  |
| 3 months | -1.08 | -1.84; -0.31 | -0.62 | -1.12; -0.12 |
| 6 months | -1.5 | -2.45; -0.55 | -1.99 | -2.59; -1.40 |
| 12 months | -1.54 | -3.40; 0.32 | -0.52 | -1.26; 0.23 |
| CAL |  |  |  |  |
| 3 months | -0.78 | -1.39; -0.16 | -0.43 | -0.92; 0.06 |
| 6 months | -0.84 | -1.80; 0.11 | -0.42 | -0.90; 0.07 |
| 12 months | -0.93 | -2.61; 0.74 | -0.17 | -0.70; 0.37 |
| BOP |  |  |  |  |
| 3 months | -0.15 | 0.64; 0.34 | -0.15 | 0.64; 0.34 |
| 6 months | -0.14 | -0.63; 0.34 | -0.14 | -0.63; 0.34 |
| 12 months | -0.31 | -0.80; 0.18 | -0.31 | -0.80; 0.18 |
| summary group | -0.1 | -0.45; 0.26 | 0.05 | -0.38; 0.47 |
