# Supplementary material for: Association between smoking cessation and periodontitis progression: a systematic review and meta-analysis of prospective observational studies
Source: Front Oral Health. 2026 Jul 29;7:1900393. doi: 10.3389/froh.2026.1900393 (PMC13461921; doi:10.3389/froh.2026.1900393)
Supplement: Supplementary file 6 [file Datasheet3.docx]

***Appendix 3.*** Domain-level reporting of the Newcastle-Ottawa Scale

| Study, year | Selection | | | | Comparability | Outcome | | | NOS Score |
| --- | --- | --- | --- | --- | --- | --- | --- | --- | --- |
|  | Representativeness of the exposed cohort | Selection of the non-exposed cohort | Ascertainment of exposure | Demonstration that outcome of interest was not present at start of study | Comparability of cases and controls on the basis of the design or analysis | Ascertainment of outcome | Was follow-up long enough for outcomes to occur （6 months） | Adequacy of follow up of cohorts |  |
| J. Bergström 2000 | 0 | 1 | 1 | 1 | 1 | 1 | 1 | 1 | 7 |
| P. M. Preshaw 2005 | 1 | 1 | 1 | 1 | 1 | 1 | 1 | 0 | 7 |
| Y. Okamoto 2006 | 1 | 1 | 1 | 1 | 1 | 1 | 1 | 1 | 8 |
| E. F. Rosa 2011 | 1 | 1 | 1 | 1 | 1 | 1 | 1 | 0 | 7 |
| M. E. Rashid 2024 | 1 | 1 | 1 | 1 | 1 | 1 | 1 | 1 | 8 |
| Arti Dixit 2024 | 1 | 1 | 1 | 1 | 1 | 1 | 1 | 1 | 8 |
