# Supplementary material for: Association between smoking cessation and periodontitis progression: a systematic review and meta-analysis of prospective observational studies
Source: Front Oral Health. 2026 Jul 29;7:1900393. doi: 10.3389/froh.2026.1900393 (PMC13461921; doi:10.3389/froh.2026.1900393)
Supplement: Supplementary file 7 [file Datasheet4.docx]

***Appendix 4.*** The exclusion reasons for all studies that were included in the full-text screening stage

| Reasons for exclusion | No |
| --- | --- |
| **Records excluded on the basis of full text screening:** | 229 |
| Non-human studies: such as animal studies (1) (2) (3) (4) (5) (6) (7-11) | 11 |
| Ineligible study population (12-14) (15, 16) (17) (18-21) (22)  Ineligible outcome (23) (24) (25) (26, 27) (28-31) (32-35) (36) (37-40) (41)  Ineligible exposure (42) (43-47) (47-50) (51-53) (54) (55-62)  Ineligible study design (63) (64) (65) (66) (67) (68) (69) (70-72) (73-80) (81) (82) (83) (84-100) (41, 101-126) (71, 127-136) (137-150) (151-154) (155, 156) (157-160) (161-172) (39, 40, 173-178) (179-182) (183) (184) (1-6, 185-191) (35, 192-194) (22, 195-201) (202-214) (26, 41, 173, 175, 178, 215-219) (139, 217, 220, 221)  Pilot study (222) (223) (224) (225) (226) (227) (228) (229) | 11  19  21  159  8 |

1.	Palmer RM, Wilson RF, Hasan AS, Scott DA. Mechanisms of action of environmental factors - Tobacco smoking. Journal of Clinical Periodontology. (2005);32(SUPPL. 6):180-95.doi: 10.1111/j.1600-051X.2005.00786.x

2.	Wan CP, Leung WK, Wong MCM, Wong RMS, Wan P, Lo ECM, et al. Effects of smoking on healing response to non-surgical periodontal therapy: A multilevel modelling analysis. Journal of Clinical Periodontology. (2009);36(3):229-39.doi: 10.1111/j.1600-051X.2008.01371.x

3.	Javed F, Tenenbaum HC, Nogueira-Filho G, Qayyum F, Correa FOB, Al-Hezaimi K, et al. Severity of periodontal disease in individuals chewing betel quid with and without tobacco. American Journal of the Medical Sciences. (2013);346(4):273-8.doi: 10.1097/MAJ.0b013e31827333fb

4.	Hasegawa T, Watase H. Multiple risk factors of periodontal disease: A study of 9260 Japanese non-smokers. Geriatrics and Gerontology International. (2004);4(1):37-43.doi: 10.1111/j.1447-0594.2003.00116.x

5.	Wiener RC. Association of smokeless tobacco use and smoking in adolescents in the United States: an analysis of data from the Youth Risk Behavior Surveillance System survey, 2011. J Am Dent Assoc. (2013);144(8):930-8.doi: 10.14219/jada.archive.2013.0212

6.	Rosa EF, Corraini P, Inoue G, Gomes EF, Guglielmetti MR, Sanda SR, et al. Effect of smoking cessation on non-surgical periodontal therapy: results after 24 months. J Clin Periodontol. (2014);41(12):1145-53.doi: 10.1111/jcpe.12313

7.	da Silva ACR, Vasques AMV, Bueno CRE, Ervolino E, Cintra LÇ, Biguetti CC, et al. Effects of cigarette smoke inhalation on the immune-inflammatory profile of experimental apical periodontitis in rats. Int Endod J. (2023);56(12):1559-70.doi: 10.1111/iej.13981

8.	da Silva ACR, Vasques AMV, Bueno CRE, Ervolino E, Cintra LTÂ, Biguetti CC, et al. Effects of cigarette smoke inhalation on the immune-inflammatory profile of experimental apical periodontitis in rats. International endodontic journal. (2023);56(12):1559-70.doi: 10.1111/iej.13981

9.	Vasques AMV, da Silva ACR, Bueno CRE, Cury MTS, Ervolino E, Cintra LTA, et al. Inflammatory profile of apical periodontitis exacerbated by cigarette smoke inhalation: Histological and immunohistochemical analysis in rats. International endodontic journal. (2023);56(4):465-74.doi: 10.1111/iej.13883

10.	Veiga Vasques AM, da Silva ACR, Bueno CRE, Duarte MAH, Ervolino E, Cintra LTA, et al. Bone Resorption in Apical Periodontitis Enhanced by Cigarette Smoke Inhalation: Histometric, Immunohistochemical, and¬†Microtomographic Analysis in Rats. J Endod. (2024);50(4):493-8.doi: 10.1016/j.joen.2024.01.005

11.	Veiga Vasques AM, da Silva ACR, Bueno CRE, Duarte MAH, Ervolino E, Cintra LTA, et al. Bone resorption in apical periodontitis enhanced by cigarette smoke inhalation: histometric, immunohistochemical and microtomographic analysis in rats. Journal of endodontics. (2024).doi: 10.1016/j.joen.2024.01.005

12.	Javed F, Al-Kheraif AA, Al Amri MD, Mikami T, Vohra F, Warnakulasuriya S, et al. Periodontal parameters and whole salivary cytokine profiles among habitual gutka chewers and non-chewers. J Periodontol. (2015);86(5):689-95.doi: 10.1902/jop.2015.140556

13.	Javed F, Vohra F, Al-Kheraif AA, Malmstrom H, Romanos GE. Comparison of periodontal inflammatory conditions among habitual gutka chewers and betel quid chewers. Oral Dis. (2015);21(4):437-42.doi: 10.1111/odi.12295

14.	Al-Askar M, Abduljabbar T, Vohra F, Pasumarthy S, Gholamiazizi E, Yunker M, et al. Comparison of self-perceived oral symptoms and periodontal parameters among habitual  shamma-chewers, gutka-chewers, and non-chewers. Quintessence Int. (2017);48(3):251-60.doi: 10.3290/j.qi.a36890

15.	Similä T, Auvinen J, Timonen M, Virtanen JI. Long-term effects of smoking on tooth loss after cessation among middle-aged Finnish adults: the Northern Finland Birth Cohort 1966 Study. BMC Public Health. (2016);16(1):867.doi: 10.1186/s12889-016-3556-1

16.	Akinkugbe AA, Sanders AE, Preisser JS, Cai J, Salazar CR, Beck JD. Environmental tobacco smoke exposure and periodontitis prevalence among nonsmokers in the hispanic community Health Study/Study of Latinos. Community Dent Oral Epidemiol. (2017);45(2):168-77.doi: 10.1111/cdoe.12275

17.	Makino A, Yamada S, Okuda K, Kato T. Nicotine involved in periodontal disease through influence on cytokine levels. FEMS Immunol Med Microbiol. (2008);52(2):282-6.doi: 10.1111/j.1574-695X.2007.00373.x

18.	Imaki M, Yoshida Y, Tanada S. Relation between smoking and periodontal disease by oral hygiene status in Japanese factory workers. Appl Human Sci. (1997);16(2):77-81.doi: 10.2114/jpa.16.77

19.	Heikkinen AM, Sorsa T, Pitkäniemi J, Tervahartiala T, Kari K, Broms U, et al. Smoking affects diagnostic salivary periodontal disease biomarker levels in adolescents. J Periodontol. (2010);81(9):1299-307.doi: 10.1902/jop.2010.090608

20.	Papantonopoulos GH. Smoking influences decision making in periodontal therapy: a retrospective clinical study. J Periodontol. (1999);70(10):1166-73.doi: 10.1902/jop.1999.70.10.1166

21.	Javali MA, Joseph B, AlQahtani NA, Khader MA, Abullias SS, AlQahtani SM. Periodontal status among adult cigarette smokers using Miswak (Salvadora persica) for oral hygiene: A cross-sectional study from Asir Region of Saudi Arabia. Tropical Journal of Pharmaceutical Research. (2020);19(10):2205-12.doi: 10.4314/tjpr.v19i10.26

22.	Al-Nazhan SA, Alsaeed SA, Al-Attas HA, Dohaithem AJ, Al-Serhan MS, Al-Maflehi NS. Prevalence of apical periodontitis and quality of root canal treatment in an adult Saudi population. Saudi Med J. (2017);38(4):413-21.doi: 10.15537/smj.2017.4.16409

23.	Hodge P, Binnie V. Smoking cessation and periodontal health--a missed opportunity? Evid Based Dent. (2009);10(1):18-9.doi: 10.1038/sj.ebd.6400632

24.	Bergström J, Eliasson S, Dock J. A 10-year prospective study of tobacco smoking and periodontal health. J Periodontol. (2000);71(8):1338-47.doi: 10.1902/jop.2000.71.8.1338

25.	Airila-Månsson S, Söder B, Jin LJ, Söder PO, Klinge B. Self-reporting of periodontal diseases and clinical assessment outcome in a Swedish urban population of smokers and non-smokers. Acta Odontol Scand. (2004);62(2):111-5.doi: 10.1080/00016350410005861

26.	Nasry HA, Preshaw PM, Stacey F, Heasman L, Swan M, Heasman PA. Smoking cessation advice for patients with chronic periodontitis. Br Dent J. (2006);200(5):272-5; discussion 65.doi: 10.1038/sj.bdj.4813307

27.	Schmidt JC, Jajjo E, Berglundh T, Zitzmann NU. Periodontitis lesions in smokers and non-smokers. Eur J Oral Sci. (2020);128(3):196-203.doi: 10.1111/eos.12693

28.	Duane B. Further evidence that periodontal bone loss increases with smoking and age. Evid Based Dent. (2014);15(3):72-3.doi: 10.1038/sj.ebd.6401038

29.	Robbins J, Ali K. How do periodontal indices compare among non-smokers, tobacco and e-cigarette smokers? Evid Based Dent. (2022);23(3):116-7.doi: 10.1038/s41432-022-0809-y

30.	Ziukaite L, Slot DE, Loos BG, Coucke W, Van der Weijden GA. Family history of periodontal disease and prevalence of smoking status among adult periodontitis patients: a cross-sectional study. Int J Dent Hyg. (2017);15(4):e28-e34.doi: 10.1111/idh.12224

31.	Javed F, Al-Zawawi AS, Allemailem KS, Almatroudi A, Mehmood A, Divakar DD, et al. Periodontal Conditions and Whole Salivary IL-17A and -23 Levels among Young Adult Cannabis sativa (Marijuana)-Smokers, Heavy Cigarette-Smokers and Non-Smokers. Int J Environ Res Public Health. (2020);17(20).doi: 10.3390/ijerph17207435

32.	Boström L, Linder LE, Bergström J. Influence of smoking on the outcome of periodontal surgery. A 5-year follow-up. J Clin Periodontol. (1998);25(3):194-201.doi: 10.1111/j.1600-051x.1998.tb02428.x

33.	Ludwick W, Massler M. Relation of dental caries experience and gingivitis to cigarette smoking in males 17 to 21 years old (at the Great Lakes Naval Training Center). J Dent Res. (1952);31(3):319-22.doi: 10.1177/00220345520310030401

34.	Kinane DF, Radvar M. The effect of smoking on mechanical and antimicrobial periodontal therapy. J Periodontol. (1997);68(5):467-72.doi: 10.1902/jop.1997.68.5.467

35.	The link between chronic smoking and periodontal disease. Dent Today. (2001);20(5):44.doi:

36.	Shah PM, Geetha RV, Thangavelu L. Prevalence of periodontitis among smokers and non-smokers. International Journal of Research in Pharmaceutical Sciences. (2019);10(2):1161-6.doi: 10.26452/ijrps.v10i2.398

37.	Rosa EF, Corraini P, de Carvalho VF, Inoue G, Gomes EF, Lotufo JP, et al. A prospective 12-month study of the effect of smoking cessation on periodontal clinical parameters. J Clin Periodontol. (2011);38(6):562-71.doi: 10.1111/j.1600-051X.2011.01723.x

38.	Thomson WM, Broadbent JM, Welch D, Beck JD, Poulton R. Cigarette smoking and periodontal disease among 32-year-olds: a prospective study of a representative birth cohort. J Clin Periodontol. (2007);34(10):828-34.doi: 10.1111/j.1600-051X.2007.01131.x

39.	da Silva RVC, Rangel TP, Corrêa MG, de Freitas Monteiro M, Casati MZ, Ruiz KG, et al. Smoking negatively impacts the clinical, microbiological, and immunological treatment response of young adults with Grade C periodontitis. J Periodontal Res. (2022);57(6):1116-26.doi: 10.1111/jre.13049

40.	Okamoto Y, Tsuboi S, Suzuki S, Nakagaki H, Ogura Y, Maeda K, et al. Effects of smoking and drinking habits on the incidence of periodontal disease and tooth loss among Japanese males: a 4-yr longitudinal study. J Periodontal Res. (2006);41(6):560-6.doi: 10.1111/j.1600-0765.2006.00907.x

41.	Machuca G, Rosales I, Lacalle JR, Machuca C, Bullón P. Effect of cigarette smoking on periodontal status of healthy young adults. J Periodontol. (2000);71(1):73-8.doi: 10.1902/jop.2000.71.1.73

42.	Zahiu SG, Fratila O. Assessment of Systemic Condition and Smoking Impact Over Incidence of Apical Periodontitis. Cureus. (2024);16(2):e55250.doi: 10.7759/cureus.55250

43.	Ahlberg J, Tuominen R, Murtomaa H. Periodontal status among male industrial workers in southern Finland with or without access to subsidized dental care. Acta Odontol Scand. (1996);54(3):166-70.doi: 10.3109/00016359609003518

44.	Goswami S, Ylöstalo P, Khan S, Knuuttila M, Bernabe E, Suominen AL. Effect of smoking on periodontal health and validation of self-reported smoking status with serum cotinine levels. Acta Odontol Scand. (2021);79(8):573-81.doi: 10.1080/00016357.2021.1917655

45.	Leow NM, Morel-Kopp MC, Woodward M, Chen Q, Tofler GH, Taylor BA. The relationship between cotinine and periodontal disease. Ann R Australas Coll Dent Surg. (2006);18:51-2.doi:

46.	Karaaslan F, Dikilitaş A, Yiğit U. The effects of vaping electronic cigarettes on periodontitis. Aust Dent J. (2020);65(2):143-9.doi: 10.1111/adj.12747

47.	Goel K, Sharma S, Baral DD, Agrawal SK. Current status of periodontitis and its association with tobacco use amongst adult population of Sunsari district, in Nepal. BMC Oral Health. (2021);21(1):66.doi: 10.1186/s12903-021-01416-3

48.	Ojima M, Hanioka T, Tanaka K, Aoyama H. Cigarette smoking and tooth loss experience among young adults: a national record linkage study. BMC Public Health. (2007);7:313.doi: 10.1186/1471-2458-7-313

49.	Rodriguez FR, Taner B, Weiger R, Walter C. Is smoking a predictor of apical periodontitis? Clin Oral Investig. (2013);17(8):1947-55.doi: 10.1007/s00784-012-0893-z

50.	Bergström J, Babcan J, Eliasson S. Tobacco smoking and dental periapical condition. Eur J Oral Sci. (2004);112(2):115-20.doi: 10.1111/j.1600-0722.2004.00112.x

51.	Madhumithaa S, Sangavi R, Sandeep AH. INFLUENCE OF TOBACCO SMOKING ON DENTAL PERIAPICAL CONDITION AMONG ADULT POPULATION-A RADIOGRAPHIC STUDY. European Journal of Molecular and Clinical Medicine. (2022);9(7):7056-65.doi:

52.	Ah MK, Johnson GK, Kaldahl WB, Patil KD, Kalkwarf KL. The effect of smoking on the response to periodontal therapy. J Clin Periodontol. (1994);21(2):91-7.doi: 10.1111/j.1600-051x.1994.tb00285.x

53.	Baumeister SE, Freuer D, Nolde M, Kocher T, Baurecht H, Khazaei Y, et al. Testing the association between tobacco smoking, alcohol consumption, and risk of periodontitis: A Mendelian randomization study. J Clin Periodontol. (2021);48(11):1414-20.doi: 10.1111/jcpe.13544

54.	Health dangers encourage periodontists to help smokers. Dent Today. (2000);19(4):32, 6.doi:

55.	Ismail AF, Ghazali AF, Daud A. Effect of electronic cigarette usage on oral health: A 6-month prospective study. Indian Journal of Public Health Research and Development. (2019);10(6):1455-60.doi: 10.5958/0976-5506.2019.01503.1

56.	Abdulkareem EH. Shisha has deleterious effects on the oral health of many! International Medical Journal. (2020);27(3):374.doi:

57.	Ashril NY, Al-Sulamani A. The effect of different types of smoking habits on periodontal attachment. J Int Acad Periodontol. (2003);5(2):41-6.doi:

58.	Lai YL, Leone CW. The relation between smoking and periodontal disease. J Mass Dent Soc. (1997);46(3):17-8, 20-2, 4.doi:

59.	Graves RC. Epidemiologic study of periodontal disease and use of tobacco. Journal of Dental Research. (1973);52(Spec.Nr):668.doi:

60.	Rizzolo D, Chiodo TA. Smokeless tobacco was to blame. Journal of Family Practice. (2008);57(4):244.doi:

61.	Paidi S, Pack AR, Thomson WM. An example of measurement and reporting of periodontal loss of attachment (LOA) in epidemiological studies: smoking and periodontal tissue destruction. N Z Dent J. (1999);95(422):118-23.doi:

62.	Oginni AO, Adeleke AA, Mejabi MO, Sotunde OA. Risk Factors for Apical Periodontitis Sub-Urban Adult Population. Niger Postgrad Med J. (2015);22(2):105-9.doi:

63.	Rashid ME, Alam MK, Akhter K, Abdelghani A, Babkair HA, Sghaireen MG. Assessing the Impact of Smoking Cessation Interventions on Periodontal Health and Gingival Inflammation in Smokers with Periodontitis. Journal of Pharmacy and Bioallied Sciences. (2024);16:S745-S7.doi: 10.4103/jpbs.jpbs_987_23

64.	Dixit A, Dhanalakshmi P, Rameshchandra PT, Chachlani KS, Dithi C, Dash KC, et al. Effect of Smoking Cessation Programs on Periodontal Disease Progression. Journal of Pharmacy and Bioallied Sciences. (2024);16:S774-S6.doi: 10.4103/jpbs.jpbs_1004_23

65.	Trullenque-Eriksson A, Derks J, Andersson JS. Onset of periodontitis - a registry-based cohort study. Clinical oral investigations. (2023);27(5):2187-95.doi: 10.1007/s00784-023-04923-5

66.	Sim KY, Jang YS, Jang YS, Nerobkova N, Park EC. Association between Smoking and Periodontal Disease in South Korean Adults. International Journal of Environmental Research and Public Health. (2023);20(5).doi: 10.3390/ijerph20054423

67.	Yeung CA. Is the use of tobacco products, especially electronic nicotine delivery systems (ENDS), associated with the incidence of oral health outcomes among US adults? Evidence-based dentistry. (2023);24(4):161-2.doi: 10.1038/s41432-023-00949-6

68.	Nupur N, Raj R. A Case Control Assessment of the Association between Kharra Chewing and Periodontal Health Status in Oral Submucous Fibrosis Patients: A Comparative Study. International Journal of Current Pharmaceutical Review and Research. (2023);15(3):46-52.doi:

69.	Arruda CV, Guilardi IJ, Pavan LMC, Greggianin BF. Oral health-related quality of life and periodontal status according to smoking status. International journal of dental hygiene. (2023).doi: 10.1111/idh.12720

70.	Yuvashri P, Renuka Devi R, Esther Nalini H, Arun Kumar Prasad P. Estimating the salivary levels of IL-35 in smokers with periodontitis: A cross sectional study. Saudi Dental Journal. (2024);36(1):168-72.doi: 10.1016/j.sdentj.2023.09.006

71.	Suragimath G, Patel JN, Mhatre TS, Ashwinirani SR, Mane DA. Effect of Smokeless Tobacco (Mishri) Application on Periodontal Health and Correlation to the Duration of Use. International Journal of Pharmaceutical Research and Allied Sciences. (2023);12(1):41-6.doi: 10.51847/MruppT7FxO

72.	Prachishree L, Panda J, Pattanayak D, Gantayat CK. IMPACT OF TOBACCO CHEWING ON ORAL HEALTH: A HOSPITAL-BASED STUDY IN EASTERN, INDIA. International Journal of Academic Medicine and Pharmacy. (2024);6(1):1788-91.doi: 10.47009/jamp.2024.6.1.353

73.	Petrauskienė S, Žemaitienė M, Bendoraitienė EA, Saldūnaitė-Mikučionienė K, Vasiliauskienė I, Zūbienė J, et al. A Cross-Sectional Study of Oral Health Status and Behavioral Risk Indicators among Non-Smoking and Currently Smoking Lithuanian Adolescents. International Journal of Environmental Research and Public Health. (2023);20(16).doi: 10.3390/ijerph20166609

74.	Nasir SM, Sultana T, Hashmi S, Ahmed M. Patterns and predictors of periodontal disease and tooth loss among users of smokeless tobacco. BMC oral health. (2023);23(1):428.doi: 10.1186/s12903-023-03087-8

75.	Mikami R, Mizutani K, Aoyama N, Matsuura T, Suda T, Takeda K, et al. Income-related inequalities in the association of smoking with periodontitis: a cross-sectional analysis in Tokyo Metropolitan Districts. Clinical oral investigations. (2023);27(2):519-28.doi: 10.1007/s00784-022-04747-9

76.	Ke L, Nogueira G, Thomson WM. Influence of case definitions on epidemiological estimates of periodontitis prevalence and its associations with smoking and OHRQoL. Community dentistry and oral epidemiology. (2023);51(2):194-200.doi: 10.1111/cdoe.12726

77.	Alqahtani SM, Gokhale ST, Elagib MFA, Shrivastava D, Nagate RR, Alshmrani BAM, et al. Assessment and Correlation of Salivary Ca, Mg, and pH in Smokers and Non-Smokers with Generalized Chronic Periodontitis. Medicina (Kaunas, Lithuania). (2023);59(4).doi: 10.3390/medicina59040765

78.	Falc√≥n-Flores JA, Jim√©nez-Corona ME, Rangel-Nieto I, Moreno-Altamirano L, Borges-Y√°√±ez SA, V√°zquez-Duran M, et al. Social determinants of health for moderate and severe periodontal disease in rural and urban populations. Community Dent Health. (2023);40(3):146-53.doi: 10.1922/CDH_00169Falcon-Flores08

79.	Arruda CV, Guilardi IJ, Pavan LMC, Greggianin BF. Oral health-related quality of life and periodontal status according to smoking status. Int J Dent Hyg. (2024);22(2):368-75.doi: 10.1111/idh.12720

80.	Amaranath BJ, Gupta S, Kumar S, Das N, Gupta I, Pratik S. Assessment of Periodontal Health Status among the Male Adult Population with a Dual Habit of Smoking and Gutkha Chewing: A Cross-Sectional Study. J Pharm Bioallied Sci. (2023);15(Suppl 2):S1020-s2.doi: 10.4103/jpbs.jpbs_246_23

81.	van der Weijden GA, de Slegte C, Timmerman MF, van der Velden U. Periodontitis in smokers and non-smokers: intra-oral distribution of pockets. J Clin Periodontol. (2001);28(10):955-60.doi: 10.1034/j.1600-051x.2001.028010955.x

82.	Jansson LE, Hagström KE. Relationship between compliance and periodontal treatment outcome in smokers. J Periodontol. (2002);73(6):602-7.doi: 10.1902/jop.2002.73.6.602

83.	Baharin B, Palmer RM, Coward P, Wilson RF. Investigation of periodontal destruction patterns in smokers and non-smokers. J Clin Periodontol. (2006);33(7):485-90.doi: 10.1111/j.1600-051X.2006.00934.x

84.	Yoshihara A, Sugita N, Yamamoto K, Kobayashi T, Hirotomi T, Ogawa H, et al. FcγRIIIb genotypes and smoking in periodontal disease progression among community-dwelling older adults in Japan. Journal of Periodontology. (2005);76(2):250-5.doi: 10.1902/jop.2005.76.2.250

85.	Santos A, Pascual A, Llopis J, Giner L, Kim DM, Levi P, et al. Self-reported Oral Hygiene Habits in Smokers and Nonsmokers Diagnosed with Periodontal Disease. Oral health & preventive dentistry. (2015);13(3):245-51.doi: 10.3290/j.ohpd.a34248

86.	Ramnath M, Shenoy N, Rao RM, Jaiswal R. Evaluation of the effect of smokeless tobacco on periodontal healthand oral hygiene status. Nitte University Journal of Health Science. (2017);7(1):52-6.doi: 10.1055/s-0040-1708696

87.	Kerdvongbundit V, Wikesjö UME. Prevalence and severity of periodontal disease at mandibular molar teeth in smokers with regular oral hygiene habits. Journal of Periodontology. (2002);73(7):735-40.doi: 10.1902/jop.2002.73.7.735

88.	Yoshida Y, Ogawa Y, Imaki M, Nakamura T, Tanada S. Lifestyles and periodontal disease of Japanese factory workers. Environ Health Prev Med. (1997);1(4):188-92.doi: 10.1007/bf02931215

89.	Wood N, Johnson RB. Use of water 'softening and conditioning systems' significantly increases the risk of periodontitis: smoking considerations. J Periodontal Res. (2004);39(6):367-72.doi: 10.1111/j.1600-0765.2004.00769.x

90.	Torrungruang K, Gongsakdi V, Laohaviraphab L, Likittanasombat K, Ratanachaiwong W. Association between cigarette smoking and the intraoral distribution of periodontal disease in Thai men over 50 years of age. J Investig Clin Dent. (2012);3(2):135-41.doi: 10.1111/j.2041-1626.2011.00105.x

91.	Tanaka K, Miyake Y, Hanioka T, Arakawa M. Active and passive smoking and prevalence of periodontal disease in young Japanese women. J Periodontal Res. (2013);48(5):600-5.doi: 10.1111/jre.12044

92.	Susin C, Albandar JM. Aggressive periodontitis in an urban population in southern Brazil. J Periodontol. (2005);76(3):468-75.doi: 10.1902/jop.2005.76.3.468

93.	Sheiham A. Periodontal disease and oral cleanliness in tobacco smokers. J Periodontol. (1971);42(5):259-63.doi: 10.1902/jop.1971.42.5.259

94.	Shariff JA, Ahluwalia KP, Papapanou PN. Relationship Between Frequent Recreational Cannabis (Marijuana and Hashish) Use and Periodontitis in Adults in the United States: National Health and Nutrition Examination Survey 2011 to 2012. J Periodontol. (2017);88(3):273-80.doi: 10.1902/jop.2016.160370

95.	Santosh ABR, J RC, Jones T, Brache M, Toro M, Tumanyan S, et al. The SmoCar Study: A Caribbean-Based Multicenter Study on the Prevalence and Disparities Linked to Smoking and Gingival Health. Int Q Community Health Educ. (2020);40(4):321-30.doi: 10.1177/0272684x19895901

96.	Pucher JJ, Shibley O, Dentino AR, Ciancio SG. Results of limited initial periodontal therapy in smokers and non-smokers. J Periodontol. (1997);68(9):851-6.doi: 10.1902/jop.1997.68.9.851

97.	Preus HR, Sandvik L, Gjermo P, Baelum V. Baseline adjustment and change revisited: effect of smoking on change in periodontal status following periodontal therapy. Eur J Oral Sci. (2014);122(2):89-99.doi: 10.1111/eos.12111

98.	Preber H, Kant T. Effect of tobacco-smoking on periodontal tissue of 15-year-old schoolchildren. J Periodontal Res. (1973);8(5):278-83.doi: 10.1111/j.1600-0765.1973.tb01116.x

99.	Preber H, Bergström J. Effect of cigarette smoking on periodontal healing following surgical therapy. J Clin Periodontol. (1990);17(5):324-8.doi: 10.1111/j.1600-051x.1990.tb01098.x

100.	Ojima M, Hanioka T, Tanaka K, Inoshita E, Aoyama H. Relationship between smoking status and periodontal conditions: findings from national databases in Japan. J Periodontal Res. (2006);41(6):573-9.doi: 10.1111/j.1600-0765.2006.00915.x

101.	Offenbacher S, Weathers DR. Effects of smokeless tobacco on the periodontal, mucosal and caries status of adolescent males. J Oral Pathol. (1985);14(2):169-81.doi: 10.1111/j.1600-0714.1985.tb00480.x

102.	Natto S, Baljoon M, Bergström J. Tobacco smoking and periodontal health in a Saudi Arabian population. J Periodontol. (2005);76(11):1919-26.doi: 10.1902/jop.2005.76.11.1919

103.	Mittal S, Dani N, Abullais SS, Al-Qahtani NA, Shah K. Effect of Smoking and Tobacco Chewing on Periodontal Disease and Non-Surgical Treatment Outcome: A Clinical and Biochemical Study. J Int Acad Periodontol. (2017);20(1):12-8.doi:

104.	Meisel P, Schwahn C, Gesch D, Bernhardt O, John U, Kocher T. Dose-effect relation of smoking and the interleukin-1 gene polymorphism in periodontal disease. J Periodontol. (2004);75(2):236-42.doi: 10.1902/jop.2004.75.2.236

105.	Meinberg TA, Canarsky-Handley AM, McClenahan AK, Poulsen DD, Marx DB, Reinhardt RA. Outcomes associated with supportive periodontal therapy in smokers and nonsmokers. J Dent Hyg. (2001);75(1):15-9.doi:

106.	Martinez-Canut P, Lorca A, Magán R. Smoking and periodontal disease severity. J Clin Periodontol. (1995);22(10):743-9.doi: 10.1111/j.1600-051x.1995.tb00256.x

107.	Markkanen H, Paunio I, Tuominen R, Rajala M. Smoking and periodontal disease in the Finnish population aged 30 years and over. J Dent Res. (1985);64(6):932-5.doi: 10.1177/00220345850640061601

108.	Luzzi LI, Greghi SL, Passanezi E, Sant'ana AC, Lauris JR, Cestari TM. Evaluation of clinical periodontal conditions in smokers and non-smokers. J Appl Oral Sci. (2007);15(6):512-7.doi: 10.1590/s1678-77572007000600011

109.	Linden GJ, Mullally BH. Cigarette smoking and periodontal destruction in young adults. J Periodontol. (1994);65(7):718-23.doi: 10.1902/jop.1994.65.7.718

110.	Kolte AP, Kolte RA, Laddha RK. Effect of smoking on salivary composition and periodontal status. J Indian Soc Periodontol. (2012);16(3):350-3.doi: 10.4103/0972-124x.100909

111.	Kerdvongbundit V, Wikesjö UM. Effect of smoking on periodontal health in molar teeth. J Periodontol. (2000);71(3):433-7.doi: 10.1902/jop.2000.71.3.433

112.	Kanmaz B, Lappin DF, Nile CJ, Buduneli N. Effects of smoking on non-surgical periodontal therapy in patients with periodontitis Stage III or IV, and Grade C. J Periodontol. (2020);91(4):442-53.doi: 10.1002/jper.19-0141

113.	Kanmaz B, Lamont G, Danacı G, Gogeneni H, Buduneli N, Scott DA. Microbiological and biochemical findings in relation to clinical periodontal status in active smokers, non-smokers and passive smokers. Tob Induc Dis. (2019);17:20.doi: 10.18332/tid/104492

114.	Jeong W, Choi DW, Kim YK, Lee HJ, Lee SA, Park EC, et al. Associations of electronic and conventional cigarette use with periodontal disease in South Korean adults. J Periodontol. (2020);91(1):55-64.doi: 10.1002/jper.19-0060

115.	Javed F, Al-Kheraif AA, Rahman I, Millan-Luongo LT, Feng C, Yunker M, et al. Comparison of Clinical and Radiographic Periodontal Status Between Habitual Water-Pipe Smokers and Cigarette Smokers. J Periodontol. (2016);87(2):142-7.doi: 10.1902/jop.2015.150235

116.	Hessari H, Vehkalahti M, Eghbal MJ, Murtomaa H. Lifelong exposure to smoking and oral health among 35- to 44-year-old Iranians. Oral Health Prev Dent. (2009);7(1):61-8.doi:

117.	Han DH, Lim S, Kim JB. The association of smoking and diabetes with periodontitis in a Korean population. J Periodontol. (2012);83(11):1397-406.doi: 10.1902/jop.2012.110686

118.	Goultschin J, Cohen HD, Donchin M, Brayer L, Soskolne WA. Association of smoking with periodontal treatment needs. J Periodontol. (1990);61(6):364-7.doi: 10.1902/jop.1990.61.6.364

119.	Feser G, Funosas E, Marí G, Escovich L. Dental death rate prevalence in smokers and non smokers adults with periodontal disease. Med Oral Patol Oral Cir Bucal. (2005);10 Suppl 2:E109-16.doi:

120.	Erdemir EO, Sonmez IS, Oba AA, Bergstrom J, Caglayan O. Periodontal health in children exposed to passive smoking. J Clin Periodontol. (2010);37(2):160-4.doi: 10.1111/j.1600-051X.2009.01510.x

121.	de Heens GL, Kikkert R, Aarden LA, van der Velden U, Loos BG. Effects of smoking on the ex vivo cytokine production in periodontitis. J Periodontal Res. (2009);44(1):28-34.doi: 10.1111/j.1600-0765.2007.01047.x

122.	BinShabaib M, SS AL, Akram Z, Khan J, Rahman I, Romanos GE, et al. Clinical periodontal status and gingival crevicular fluid cytokine profile among cigarette-smokers, electronic-cigarette users and never-smokers. Arch Oral Biol. (2019);102:212-7.doi: 10.1016/j.archoralbio.2019.05.001

123.	Bibars AR, Obeidat SR, Khader Y, Mahasneh AM, Khabour OF. The Effect of Waterpipe Smoking on Periodontal Health. Oral Health Prev Dent. (2015);13(3):253-9.doi: 10.3290/j.ohpd.a32671

124.	Bergström J, Preber H. The influence of cigarette smoking on the development of experimental gingivitis. J Periodontal Res. (1986);21(6):668-76.doi: 10.1111/j.1600-0765.1986.tb01504.x

125.	Bergström J, Eliasson S, Dock J. Exposure to tobacco smoking and periodontal health. J Clin Periodontol. (2000);27(1):61-8.doi: 10.1034/j.1600-051x.2000.027001061.x

126.	Bergström J. Tobacco smoking and risk for periodontal disease. J Clin Periodontol. (2003);30(2):107-13.doi: 10.1034/j.1600-051x.2003.00272.x

127.	Nakayama Y, Mori M. The relationship between periodontal disease and oral health behavior in adult Japanese people. Sapporo Medical journal. (2012);81(1-6):13-9.doi:

128.	Minaya-Sánchez M, Medina-Solís CE, Maupomé G, Vallejos-Sánchez AA, Casanova-Rosado JF, Marquez-Corona MDL. Prevalence of and risk indicators for chronic periodontitis in males from Campeche, Mexico. Revista de Salud Publica. (2007);9(3):388-98.doi: 10.1590/S0124-00642007000300007

129.	Gomes SC, Piccinin FB, Oppermann RV, Susin C, Nonnenmacher CI, Mutters R, et al. Periodontal status in smokers and never-smokers: Clinical findings and real-time polymerase chain reaction quantification of putative periodontal pathogens. Journal of Periodontology. (2006);77(9):1483-90.doi: 10.1902/jop.2006.060026

130.	Amarasena N, Ikeda N, Win KKS, Yamaguchi Y, Takehara T, Miyazaki H. Factors associated with severe periodontitis in rural Cambodia. Asia-Pacific Journal of Public Health. (2004);16(1):50-3.doi: 10.1177/101053950401600109

131.	Ylöstalo P, Sakki T, Laitinen J, Järvelin MR, Knuuttila M. The relation of tobacco smoking to tooth loss among young adults. Eur J Oral Sci. (2004);112(2):121-6.doi: 10.1111/j.0909-8836.2004.00111.x

132.	Wickholm S, Söder PO, Galanti MR, Söder B, Klinge B. Periodontal disease in a group of Swedish adult snuff and cigarette users. Acta Odontol Scand. (2004);62(6):333-8.doi: 10.1080/00016350410001801

133.	Vora MV, Chaffee BW. Tobacco-use patterns and self-reported oral health outcomes: A cross-sectional assessment of the Population Assessment of Tobacco and Health study, 2013-2014. J Am Dent Assoc. (2019);150(5):332-44.e2.doi: 10.1016/j.adaj.2018.12.004

134.	Torrungruang K, Nisapakultorn K, Sutdhibhisal S, Tamsailom S, Rojanasomsith K, Vanichjakvong O, et al. The effect of cigarette smoking on the severity of periodontal disease among older Thai adults. J Periodontol. (2005);76(4):566-72.doi: 10.1902/jop.2005.76.4.566

135.	Tomar SL, Asma S. Smoking-Attributable Periodontitis in the United States: Findings From NHANES III. J Periodontol. (2000);71(5):743-51.doi: 10.1902/jop.2000.71.5.743

136.	Sutton JD, Salas Martinez ML, Gerkovich MM. Environmental Tobacco Smoke and Periodontitis in United States Non-Smokers, 2009 to 2012. J Periodontol. (2017);88(6):565-74.doi: 10.1902/jop.2017.160725

137.	Susin C, Haas AN, Valle PM, Oppermann RV, Albandar JM. Prevalence and risk indicators for chronic periodontitis in adolescents and young adults in south Brazil. J Clin Periodontol. (2011);38(4):326-33.doi: 10.1111/j.1600-051X.2011.01699.x

138.	Stoltenberg JL, Osborn JB, Pihlstrom BL, Herzberg MC, Aeppli DM, Wolff LF, et al. Association between cigarette smoking, bacterial pathogens, and periodontal status. J Periodontol. (1993);64(12):1225-30.doi: 10.1902/jop.1993.64.12.1225

139.	SSY AL, Natto ZS, Midle JB, Gyurko R, O'Neill R, Steffensen B. Association between time since quitting smoking and periodontitis in former smokers in the National Health and Nutrition Examination Surveys (NHANES) 2009 to 2012. J Periodontol. (2019);90(1):16-25.doi: 10.1002/jper.18-0183

140.	Sopińska K, Bołtacz-Rzepkowska E. The influence of tobacco smoking on dental periapical condition in a sample of an adult population of the Łódź region, Poland. Int J Occup Med Environ Health. (2020);33(1):45-57.doi: 10.13075/ijomeh.1896.01460

141.	Segura-Egea JJ, Jiménez-Pinzón A, Ríos-Santos JV, Velasco-Ortega E, Cisneros-Cabello R, Poyato-Ferrera MM. High prevalence of apical periodontitis amongst smokers in a sample of Spanish adults. Int Endod J. (2008);41(4):310-6.doi: 10.1111/j.1365-2591.2007.01365.x

142.	Schenkein HA, Gunsolley JC, Koertge TE, Schenkein JG, Tew JG. Smoking and its effects on early-onset periodontitis. J Am Dent Assoc. (1995);126(8):1107-13.doi: 10.14219/jada.archive.1995.0327

143.	Sanders A, Slade G. State cigarette excise tax, secondhand smoke exposure, and periodontitis in US nonsmokers. Am J Public Health. (2013);103(4):740-6.doi: 10.2105/ajph.2011.300579

144.	Rösing CK, Gomes SC, Carvajal P, Gómez M, Costa R, Toledo A, et al. Impact of smoking on gingival inflammation in representative samples of three South American cities. Braz Oral Res. (2019);33:e090.doi: 10.1590/1807-3107bor-2019.vol33.0090

145.	Ragghianti MS, Greghi SL, Lauris JR, Sant'ana AC, Passanezi E. Influence of age, sex, plaque and smoking on periodontal conditions in a population from Bauru, Brazil. J Appl Oral Sci. (2004);12(4):273-9.doi: 10.1590/s1678-77572004000400004

146.	Pham TAV, Kieu TQ, Ngo LTQ. Risk factors of periodontal disease in Vietnamese patients. J Investig Clin Dent. (2018);9(1).doi: 10.1111/jicd.12272

147.	Peršić Bukmir R, Jurčević Grgić M, Brumini G, Spalj S, Pezelj-Ribaric S, Brekalo Pršo I. Influence of tobacco smoking on dental periapical condition in a sample of Croatian adults. Wien Klin Wochenschr. (2016);128(7-8):260-5.doi: 10.1007/s00508-015-0910-8

148.	Morita I, Sheiham A, Nakagaki H, Yoshii S, Mizuno K, Sabbah W. Is there a relationship between periodontal disease and smoking after adjusting for job classification in Japanese employed males? Oral Health Prev Dent. (2011);9(1):83-9.doi:

149.	Moimaz SA, Zina LG, Saliba O, Garbin CA. Smoking and periodontal disease: clinical evidence for an association. Oral Health Prev Dent. (2009);7(4):369-76.doi:

150.	Mahapatra S, Chaly PE, Mohapatra SC, Madhumitha M. Influence of tobacco chewing on oral health: A hospital-based cross-sectional study in Odisha. Indian J Public Health. (2018);62(4):282-6.doi: 10.4103/ijph.IJPH_327_17

151.	Lee M, Choi YH, Sagong J, Yu S, Kim Y, Lee D, et al. The interactive association of smoking and drinking levels with presence of periodontitis in South Korean adults. BMC Oral Health. (2016);16(1):80.doi: 10.1186/s12903-016-0268-y

152.	Kulkarni V, Uttamani JR, Bhatavadekar NB. Comparison of clinical periodontal status among habitual smokeless-tobacco users and cigarette smokers. Int Dent J. (2016);66(1):29-35.doi: 10.1111/idj.12192

153.	Jogezai U, Maxood A, Khan NA. Comparison of periodontal health status of smokers versus non-smokers. J Ayub Med Coll Abbottabad. (2013);25(1-2):183-6.doi:

154.	Heikkinen AM, Pajukanta R, Pitkäniemi J, Broms U, Sorsa T, Koskenvuo M, et al. The effect of smoking on periodontal health of 15- to 16-year-old adolescents. J Periodontol. (2008);79(11):2042-7.doi: 10.1902/jop.2008.080205

155.	Haber J, Wattles J, Crowley M, Mandell R, Joshipura K, Kent RL. Evidence for cigarette smoking as a major risk factor for periodontitis. J Periodontol. (1993);64(1):16-23.doi: 10.1902/jop.1993.64.1.16

156.	Gopalankutty N, Vadakkekuttical RJ, Remadevi S, Pillai AS. Prevalence of periodontitis and its correlates among tribal population of Attapady block, Palakkad District, Kerala. J Indian Soc Periodontol. (2020);24(3):264-70.doi: 10.4103/jisp.jisp_248_19

157.	Do LG, Slade GD, Roberts-Thomson KF, Sanders AE. Smoking-attributable periodontal disease in the Australian adult population. J Clin Periodontol. (2008);35(5):398-404.doi: 10.1111/j.1600-051X.2008.01223.x

158.	Do GL, Spencer AJ, Roberts-Thomson K, Ha HD. Smoking as a risk indicator for periodontal disease in the middle-aged Vietnamese population. Community Dent Oral Epidemiol. (2003);31(6):437-46.doi: 10.1046/j.1600-0528.2003.00009.x

159.	Chu YH, Tatakis DN, Wee AG. Smokeless tobacco use and periodontal health in a rural male population. J Periodontol. (2010);81(6):848-54.doi: 10.1902/jop.2010.090310

160.	Carasol M, Llodra JC, Fernandez-Meseguer A, Bravo M, Garcia-Margallo MT, Calvo-Bonacho E, et al. Periodontal conditions among employed adults in Spain. J Clin Periodontol. (2016);43(7):548-56.doi: 10.1111/jcpe.12558

161.	Beklen A, Sali N, Yavuz MB. The impact of smoking on periodontal status and dental caries. Tob Induc Dis. (2022);20:72.doi: 10.18332/tid/152112

162.	Arowojolu MO, Fawole OI, Dosumu EB, Opeodu OI. A comparative study of the oral hygiene status of smokers and non-smokers in Ibadan, Oyo state. Niger Med J. (2013);54(4):240-3.doi: 10.4103/0300-1652.119627

163.	Arbes SJ, Jr., Agústsdóttir H, Slade GD. Environmental tobacco smoke and periodontal disease in the United States. Am J Public Health. (2001);91(2):253-7.doi: 10.2105/ajph.91.2.253

164.	Anand PS, Mishra S, Nagle D, Kamath NP, Kamath KP, Anil S. Patterns of Periodontal Destruction among Smokeless Tobacco Users in a Central Indian Population. Healthcare (Basel). (2021);9(6).doi: 10.3390/healthcare9060744

165.	Anand PS, Kamath KP, Shekar BR, Anil S. Relationship of smoking and smokeless tobacco use to tooth loss in a central Indian population. Oral Health Prev Dent. (2012);10(3):243-52.doi:

166.	Amarasena N, Ekanayaka AN, Herath L, Miyazaki H. Tobacco use and oral hygiene as risk indicators for periodontitis. Community Dent Oral Epidemiol. (2002);30(2):115-23.doi: 10.1034/j.1600-0528.2002.300205.x

167.	AlQobaly L, Abed H, Alsahafi Y, Sabbah W, Hakeem FF. Does smoking explain the association between use of e-cigarettes and self-reported periodontal disease? J Dent. (2022);122:104164.doi: 10.1016/j.jdent.2022.104164

168.	Al-Habashneh R, Al-Omari MA, Taani DQ. Smoking and caries experience in subjects with various form of periodontal diseases from a teaching hospital clinic. Int J Dent Hyg. (2009);7(1):55-61.doi: 10.1111/j.1601-5037.2008.00349.x

169.	Haber J, Kent RL. Cigarette smoking in a periodontal practice. J Periodontol. (1992);63(2):100-6.doi: 10.1902/jop.1992.63.2.100

170.	Calsina G, Ramón JM, Echeverría JJ. Effects of smoking on periodontal tissues. J Clin Periodontol. (2002);29(8):771-6.doi: 10.1034/j.1600-051x.2002.290815.x

171.	Bergström J, Floderus-Myrhed B. Co-twin control study of the relationship between smoking and some periodontal disease factors. Community Dent Oral Epidemiol. (1983);11(2):113-6.doi: 10.1111/j.1600-0528.1983.tb01367.x

172.	Al-Wahadni A, Linden GJ. The effects of cigarette smoking on the periodontal condition of young Jordanian adults. J Clin Periodontol. (2003);30(2):132-7.doi: 10.1034/j.1600-051x.2003.00279.x

173.	Preshaw PM, Heasman L, Stacey F, Steen N, McCracken GI, Heasman PA. The effect of quitting smoking on chronic periodontitis. J Clin Periodontol. (2005);32(8):869-79.doi: 10.1111/j.1600-051X.2005.00779.x

174.	Nishida N, Yamamoto Y, Tanaka M, Kataoka K, Kuboniwa M, Nakayama K, et al. Association between involuntary smoking and salivary markers related to periodontitis: a 2-year longitudinal study. J Periodontol. (2008);79(12):2233-40.doi: 10.1902/jop.2008.080149

175.	Kibayashi M, Tanaka M, Nishida N, Kuboniwa M, Kataoka K, Nagata H, et al. Longitudinal study of the association between smoking as a periodontitis risk and salivary biomarkers related to periodontitis. J Periodontol. (2007);78(5):859-67.doi: 10.1902/jop.2007.060292

176.	Ju X, Mejia G, Chrisopoulos S, Luzzi L, Jamieson LM. A longitudinal assessment of chronic periodontitis in Australian adults. J Clin Periodontol. (2023);50(2):276-85.doi: 10.1111/jcpe.13741

177.	Albandar JM, Streckfus CF, Adesanya MR, Winn DM. Cigar, pipe, and cigarette smoking as risk factors for periodontal disease and tooth loss. J Periodontol. (2000);71(12):1874-81.doi: 10.1902/jop.2000.71.12.1874

178.	Akram Z, Aati S, Alrahlah A, Vohra F, Fawzy A. Longitudinal evaluation of clinical, spectral and tissue degradation biomarkers in progression of periodontitis among cigarette and electronic cigarette smokers. J Dent. (2021);109:103678.doi: 10.1016/j.jdent.2021.103678

179.	Morse DE, Avlund K, Christensen LB, Fiehn NE, Molbo D, Holmstrup P, et al. Smoking and drinking as risk indicators for tooth loss in middle-aged Danes. J Aging Health. (2014);26(1):54-71.doi: 10.1177/0898264313509278

180.	Fisher MA, Taylor GW, Tilashalski KR. Smokeless tobacco and severe active periodontal disease, NHANES III. J Dent Res. (2005);84(8):705-10.doi: 10.1177/154405910508400804

181.	Yanagisawa T, Ueno M, Shinada K, Ohara S, Wright FA, Kawaguchi Y. Relationship of smoking and smoking cessation with oral health status in Japanese men. J Periodontal Res. (2010);45(2):277-83.doi: 10.1111/j.1600-0765.2009.01233.x

182.	Ueno M, Ohara S, Sawada N, Inoue M, Tsugane S, Kawaguchi Y. The association of active and secondhand smoking with oral health in adults: Japan public health center-based study. Tob Induc Dis. (2015);13(1):19.doi: 10.1186/s12971-015-0047-6

183.	Gugnani N, Gugnani S. Can smoking cessation impact the incidence and progression of periodontitis? Evid Based Dent. (2020);21(4):122-3.doi: 10.1038/s41432-020-0136-0

184.	Solomon HA, Priore RL, Bross ID. Cigarette smoking and periodontal disease. J Am Dent Assoc. (1968);77(5):1081-4.doi: 10.14219/jada.archive.1968.0331

185.	Johannsen A, Asberg M, Soder PO, Soder B. Anxiety, gingival inflammation and periodontal disease in non-smokers and smokers - an epidemiological study. J Clin Periodontol. (2005);32(5):488-91.doi: 10.1111/j.1600-051X.2005.00704.x

186.	Picard JP, Singer DL, Kells L, Fisher S, Lix L, Scott DA. Variation in tobacco use profiles obtained from periodontal maintenance patients. J Dent. (2007);35(12):934-8.doi: 10.1016/j.jdent.2007.09.007

187.	Dietrich T, Maserejian NN, Joshipura KJ, Krall EA, Garcia RI. Tobacco use and incidence of tooth loss among US male health professionals. J Dent Res. (2007);86(4):373-7.doi: 10.1177/154405910708600414

188.	Kenney EB, Kraal JH, Saxe SR, Jones J. The effect of cigarette smoke on human oral polymorphonuclear leukocytes. J Periodontal Res. (1977);12(4):227-34.doi: 10.1111/j.1600-0765.1977.tb00126.x

189.	Boulaamaim T, Tenenbaum H, Davideau JL, Huck O. Selective Influence of Smoking on Periodontal Treatment Outcomes after 3 Years of Follow-up. Oral Health Prev Dent. (2020);18(1):823-31.doi: 10.3290/j.ohpd.a45087

190.	Rolandsson M, Hellqvist L, Lindqvist L, Hugoson A. Effects of snuff on the oral health status of adolescent males: a comparative study. Oral Health Prev Dent. (2005);3(2):77-85.doi:

191.	Johnson GK, Guthmiller JM. The impact of cigarette smoking on periodontal disease and treatment. Periodontol 2000. (2007);44:178-94.doi: 10.1111/j.1600-0757.2007.00212.x

192.	Patturaja K, Rajasekar A, Nasim I. Impact of smoking on periodontal health. European Journal of Molecular and Clinical Medicine. (2020);7(1):1099-104.doi:

193.	Chauncey HH, Baric JM. Effect of cigarette smoking on periodontal disease variables in a normal, aging male population. Gerontologist. (1981);21(Spec. Iss.):211.doi:

194.	Ctri. Indicators of Gum Diseases. <https://trialsearchwhoint/Trial2aspx?TrialID=CTRI/2018/11/016360>. (2018).doi:

195.	Chatzopoulos G. Smoking, Smokeless Tobacco, and Alcohol Consumption as Contributing Factors to Periodontal Disease. Northwest Dent. (2016);95(1):37-41.doi:

196.	Salem A, Hilow H, Khraisat A, Smadi L, Ryalat S. Association between intensity of smoking and periodontal pockets among young university students. Odontostomatol Trop. (2008);31(122):5-10.doi:

197.	Nazaryan R, Kryvenko L, Zakut Y, Karnaukh O, Gargin V. Application of estimated oral health indices in adolescents with tobacco addiction. Pol Merkur Lekarski. (2020);48(287):327-30.doi:

198.	Hamel SB, Craig BJ. The effects of cigarette smoking on periodontal disease. Probe. (1997);31(6):204-6.doi:

199.	Affrin JH. Assessment of oral hygiene status in smokers, non smokers and alcoholics. Research Journal of Pharmacy and Technology. (2015);8(8):1167-70.doi: 10.5958/0974-360X.2015.00211.5

200.	Demirayak M, Seker BK. Effects of smoking on periodontal health status: a retrospective study. Tobacco induced diseases. (2018);16:34‐.doi: 10.18332/tid/94787

201.	Johannsen A, Wickholm S, Andersson P. Tobacco cessation interventions by Swedish dental hygienists--a questionnaire study. Swed Dent J. (2012);36(1):45-52.doi:

202.	Gopalakrishnan D, Miller PD, Mahuli AV, Sangamithra S, Phantumvanit P, Buranawat B. Prospective evaluation of periodontally diseased molars in smokers using the Miller-McEntire Periodontal Prognostic Index. J Indian Soc Periodontol. (2018);22(4):304-9.doi: 10.4103/jisp.jisp_272_18

203.	Meenawat A, Govila V, Goel S, Verma S, Punn K, Srivastava V, et al. Evaluation of the effect of nicotine and metabolites on the periodontal status and the mRNA expression of interleukin-1β in smokers with chronic periodontitis. J Indian Soc Periodontol. (2015);19(4):381-7.doi: 10.4103/0972-124x.157879

204.	Rifai M, Aoun G, Majzoub Z. Evaluation of the Papillary Gingival Vasculature in Smokers and Nonsmokers with Chronic Periodontitis: A Clinical In Vivo Study. J Int Soc Prev Community Dent. (2020);10(3):368-75.doi: 10.4103/jispcd.JISPCD_134_20

205.	Han JH, Jeong SN, Lee JH. A retrospective epidemiological investigation of periodontitis risk and current smoking status based on the number of cigarettes per day and the Fagerström Test for Nicotine Dependence: a preliminary pilot study. J Periodontal Implant Sci. (2022).doi: 10.5051/jpis.2202560128

206.	Bergström J, Eliasson S. Noxious effect of cigarette smoking on periodontal health. J Periodontal Res. (1987);22(6):513-7.doi: 10.1111/j.1600-0765.1987.tb02364.x

207.	Kraal JH, Kenney EB. The response of polymorphonuclear leukocytes to chemotactic stimulation for smokers and non-smokers. J Periodontal Res. (1979);14(5):383-9.doi: 10.1111/j.1600-0765.1979.tb00235.x

208.	Peruzzo DC, Gimenes JH, Taiete T, Casarin RC, Feres M, Sallum EA, et al. Impact of smoking on experimental gingivitis. A clinical, microbiological and immunological prospective study. J Periodontal Res. (2016);51(6):800-11.doi: 10.1111/jre.12363

209.	Faddy MJ, Cullinan MP, Palmer JE, Westerman B, Seymour GJ. Ante-dependence modeling in a longitudinal study of periodontal disease: the effect of age, gender, and smoking status. J Periodontol. (2000);71(3):454-9.doi: 10.1902/jop.2000.71.3.454

210.	Feldman RS, Bravacos JS, Rose CL. Association between smoking different tobacco products and periodontal disease indexes. J Periodontol. (1983);54(8):481-7.doi: 10.1902/jop.1983.54.8.481

211.	Fisher S, Kells L, Picard JP, Gelskey SC, Singer DL, Lix L, et al. Progression of periodontal disease in a maintenance population of smokers and non-smokers: a 3-year longitudinal study. J Periodontol. (2008);79(3):461-8.doi: 10.1902/jop.2008.070296

212.	Kaldahl WB, Johnson GK, Patil KD, Kalkwarf KL. Levels of cigarette consumption and response to periodontal therapy. J Periodontol. (1996);67(7):675-81.doi: 10.1902/jop.1996.67.7.675

213.	Wimmer G, Köhldorfer G, Mischak I, Lorenzoni M, Kallus KW. Coping with stress: its influence on periodontal therapy. J Periodontol. (2005);76(1):90-8.doi: 10.1902/jop.2005.76.1.90

214.	Zeng J, Williams SM, Fletcher DJ, Cameron CM, Broadbent JM, Shearer DM, et al. Reexamining the association between smoking and periodontitis in the dunedin study with an enhanced analytical approach. J Periodontol. (2014);85(10):1390-7.doi: 10.1902/jop.2014.130577

215.	Leite FRM, López R, Pajaniaye JB, Nascimento GG. Effect of Smoking Exposure on Nonsurgical Periodontal Therapy: 1-Year Follow-up. J Dent Res. (2023);102(3):280-6.doi: 10.1177/00220345221135100

216.	Costa FO, Cota LOM. Cumulative smoking exposure and cessation associated with the recurrence of periodontitis in periodontal maintenance therapy: A 6-year follow-up. J Periodontol. (2019);90(8):856-65.doi: 10.1002/jper.18-0635

217.	Chaffee BW, Couch ET, Vora MV, Holliday RS. Oral and periodontal implications of tobacco and nicotine products. Periodontol 2000. (2021);87(1):241-53.doi: 10.1111/prd.12395

218.	Eke PI, Thornton-Evans GO, Wei L, Borgnakke WS, Dye BA, Genco RJ. Periodontitis in US Adults: National Health and Nutrition Examination Survey 2009-2014. J Am Dent Assoc. (2018);149(7):576-88.e6.doi: 10.1016/j.adaj.2018.04.023

219.	Johnson GK, Hill M. Cigarette smoking and the periodontal patient. J Periodontol. (2004);75(2):196-209.doi: 10.1902/jop.2004.75.2.196

220.	Pihlstrom BL, Michalowicz BS, Johnson NW. Periodontal diseases. Lancet. (2005);366(9499):1809-20.doi: 10.1016/s0140-6736(05)67728-8

221.	Apatzidou DA. The role of cigarette smoking in periodontal disease and treatment outcomes of dental implant therapy. Periodontol 2000. (2022);90(1):45-61.doi: 10.1111/prd.12449

222.	Shereef M, Sanara PP, Karuppanan S, Noorudeen AM, Joseph K. The effect of cigarette smoking on the severity of periodontal diseases among adults of Kothamangalam Town, Kerala. J Pharm Bioallied Sci. (2015);7(Suppl 2):S648-51.doi: 10.4103/0975-7406.163588

223.	Ng SK, Leung WK. A community study of periodontal attachment loss in smokers. Int Dent J. (2008);58(5):243-6.doi: 10.1111/j.1875-595x.2008.tb00195.x

224.	Kropff B, Petersilka G, Flemmig T, Ehmke B, Heuft G, Schneider G. Success of a smoking cessation programme in smoking behaviour of chronic periodontitis patients and identification of predictors of motivation for smoking cessation - a pilot study. Int J Dent Hyg. (2016);14(3):168-77.doi: 10.1111/idh.12124

225.	Javed F, Abduljabbar T, Vohra F, Malmstrom H, Rahman I, Romanos GE. Comparison of Periodontal Parameters and Self-Perceived Oral Symptoms Among Cigarette Smokers, Individuals Vaping Electronic Cigarettes, and Never-Smokers. J Periodontol. (2017);88(10):1059-65.doi: 10.1902/jop.2017.170197

226.	Cho YD, Kim PJ, Kim HG, Seol YJ, Lee YM, Ku Y, et al. Transcriptomics and methylomics in chronic periodontitis with tobacco use: a pilot study. Clin Epigenetics. (2017);9:81.doi: 10.1186/s13148-017-0381-z

227.	Al Kawas S, Al-Marzooq F, Rahman B, Shearston JA, Saad H, Benzina D, et al. The impact of smoking different tobacco types on the subgingival microbiome and periodontal health: a pilot study. Sci Rep. (2021);11(1):1113.doi: 10.1038/s41598-020-80937-3

228.	Han JH, Jeong SN, Lee JH. A retrospective epidemiological investigation of periodontitis risk and current smoking status based on the number of cigarettes per day and the Fagerstr√∂m Test for Nicotine Dependence: a preliminary pilot study. J Periodontal Implant Sci. (2023);53(2):135-44.doi: 10.5051/jpis.2202560128

229.	Wadia R, Booth V, Yap HF, Moyes DL. A pilot study of the gingival response when smokers switch from smoking to vaping. Br Dent J. (2016);221(11):722-6.doi: 10.1038/sj.bdj.2016.914
