# Supplementary material for: Association between smoking cessation and periodontitis progression: a systematic review and meta-analysis of prospective observational studies
Source: Front Oral Health. 2026 Jul 29;7:1900393. doi: 10.3389/froh.2026.1900393 (PMC13461921; doi:10.3389/froh.2026.1900393)
Supplement: Supplementary file 8 [file Datasheet5.docx]

***Appendix 5.* GRADE approach**

| **Outcomes** | **Follow-up duration（month）** | **Certainty assessment** | | | | | | | **No of patients** | | **Certainty** |
| --- | --- | --- | --- | --- | --- | --- | --- | --- | --- | --- | --- |
|  |  | **No of studies** | **Study design** | **Risk of bias** | **Inconsistency** | **Indirectness** | **Imprecision** | **Other considerations** | **Intervention** | **Control** |  |
| Probing depth | 3 | 3 | non-randomised studies | not serious | serious^a^ | not serious | serious^b^ | none | 226 | 245 | ⨁◯◯◯ Very low^a,b^ |
|  | 6 | 4 | non-randomised studies | not serious | serious^a^ | not serious | serious^b^ | none | 326 | 345 | ⨁◯◯◯ Very low^a,b^ |
|  | 12 | 3 | non-randomised studies | not serious | serious^a^ | not serious | serious^b^ | none | 226 | 245 | ⨁◯◯◯ Very low^a,b^ |
| Clinical attachment level | 3 | 3 | non-randomised studies | not serious | serious^a^ | not serious | very serious^b,c^ | none | 227 | 245 | ⨁◯◯◯ Very low^a,b,c^ |
|  | 6 | 3 | non-randomised studies | not serious | serious^a^ | not serious | very serious^b,c^ | none | 227 | 245 | ⨁◯◯◯ Very low^a,b,c^ |
|  | 12 | 3 | non-randomised studies | not serious | serious^a^ | not serious | very serious^b,c^ | none | 227 | 245 | ⨁◯◯◯ Very low^a,b,c^ |
| Bleeding on probing | 3 | 2 | non-randomised studies | not serious | not serious | not serious | very serious^b,c^ | none | 26 | 45 | ⨁◯◯◯ Very low^b,c^ |
|  | 6 | 2 | non-randomised studies | not serious | not serious | not serious | very serious^b,c^ | none | 26 | 45 | ⨁◯◯◯ Very low^b,c^ |
|  | 12 | 2 | non-randomised studies | not serious | not serious | not serious | very serious^b,c^ | none | 26 | 45 | ⨁◯◯◯ Very low^b,c^ |
|  | Summary group (longest follow-up duration was 10 years) | 3 | non-randomised studies | not serious | not serious | not serious | very serious^b,c^ | none | 66 | 73 | ⨁◯◯◯ Very low^b,c^ |
| Plaque index | 3 | 2 | non-randomised studies | not serious | serious^a^ | not serious | very serious^b,c^ | none | 240 | 228 | ⨁◯◯◯ Very low^a,b,c^ |
|  | 6 | 2 | non-randomised studies | not serious | serious^a^ | not serious | very serious^b,c^ | none | 240 | 228 | ⨁◯◯◯ Very low^a,b,c^ |
|  | 9 | 2 | non-randomised studies | not serious | serious^a^ | not serious | very serious^b,c^ | none | 240 | 228 | ⨁◯◯◯ Very low^a,b,c^ |
|  | 12 | 2 | non-randomised studies | not serious | serious^a^ | not serious | very serious^b,c^ | none | 240 | 228 | ⨁◯◯◯ Very low^a,b,c^ |

CI: confidence interval

**Explanations**

a. Higher heterogeneity

b. N < 2000

c. CI cross the clinical decision threshold between recommending and not recommending treatment
