# Supplementary material for: Association between smoking cessation and periodontitis progression: a systematic review and meta-analysis of prospective observational studies
Source: Front Oral Health. 2026 Jul 29;7:1900393. doi: 10.3389/froh.2026.1900393 (PMC13461921; doi:10.3389/froh.2026.1900393)
Supplement: Supplementary file 14 [file Datasheet7.docx]

***Appendix 7***. Sensitivity analysis for CAL A. 3-month group. B. 6-month group. C. 12-month group
